# Formin 3 stabilizes the cytoskeleton of *Drosophila* tendon cells, thus enabling them to resist muscle tensile forces

**DOI:** 10.1242/jcs.263543

**Published:** 2025-04-15

**Authors:** Helena Pissarek, Na Huang, Leanna H. Frasch, Hermann Aberle, Manfred Frasch

**Affiliations:** ^1^ Friedrich-Alexander-Universität Erlangen-Nürnberg, Department of Biology, Division of Developmental Biology, Staudtstr. 5, 91058 Erlangen, Germany; ^2^ Heinrich Heine University Düsseldorf, Department of Biology, Institute for Functional Cell Morphology, Universitätsstr. 1, 40225 Düsseldorf, Germany

**Keywords:** Formin, Cytoskeleton, F-actin, Microtubules, Tendon cells, Muscle attachment

## Abstract

The cytoskeleton of *Drosophila* tendon cells features specialized F-actin and microtubule arrays that endow these cells with resistance to the tensile forces exerted by the attached muscles. In a forward genetic screen for mutants with neuromuscular junction and muscle morphology phenotypes in larvae, we identified *formin 3* (*form3*) as a crucial component for stabilizing these cytoskeletal arrays under muscle tension. *form3* mutants exhibit severely stretched tendon cells in contact with directly attached larval body wall muscles, leading to muscle retraction and rounding. Both the actomyosin and microtubule arrays are expanded likewise in these mutants and can separate laterally in extreme cases. Analysis of a natively HA-tagged, functional version of Form3 reveals that Form3 is distributed along the length of these cytoskeletal arrays. Based on our findings and existing data on vertebrate and *Caenorhabditis elegans* orthologs of *form3*, we propose that the primary function of Form3 in this context is to co-bundle actin filaments and microtubules, thus maximizing the rigidity of these cytoskeletal structures against muscle tensile forces.

## Teaser

**Highlighted Article:** The disrupted cytoskeletal arrays within tendon cells in *formin 3* mutants, along with the mid-filament localization of Formin 3, provide further insight into the specific actions of inverted formins.

## INTRODUCTION

The cytoskeleton of animal cells is tailored intricately to meet the specific physiological functions and mechanical requirements of each type of cell. A case in point is the cytoskeleton in the tendon cells of *Drosophila melanogaster*, which transmits strong muscle forces, such as those from larval body wall and adult indirect flight muscles, to the rigid exoskeleton provided by the cuticle (Schweitzer et al., 2010; Maartens and Brown, 2015). *Drosophila* tendon cells represent specific epidermal cells that are programmed by the transcription factor Stripe and its downstream effectors. Bi-directional cross talk between muscles and tendon cells promotes differentiation of the myotendinous junctions (MTJs), myotube pathfinding and formation of stable muscle attachments able to withstand the contractile forces of attached muscles (Becker et al., 1997; Yarnitzky et al., 1997; Schweitzer et al., 2010; Ordan and Volk, 2015). Many aspects of the development, cellular features and molecular components of these MTJs (also referred to as muscle attachment sites, MASs) have been investigated in embryos and larvae (Maartens and Brown, 2015). In addition, some specific features have been studied in indirect flight muscle attachments to epithelial tendon cells in the adult notum (Reedy and Beall, 1993; Weitkunat et al., 2014; Valdivia et al., 2017).

In larvae, two types of muscle attachments can be distinguished: direct and indirect (Maartens and Brown, 2015). Although the two types share most of their cellular and molecular features, direct MASs involve a single muscle attaching to a single tendon cell at each end, which is typical for MASs between segment borders (intrasegmental). By contrast, indirect MASs are characterized by the attachment of three cells: the ends of two muscles and a tendon cell, a configuration found at segment borders (intersegmental) (Prokop et al., 1998a; Maartens and Brown, 2015).

**Fig. 1.**
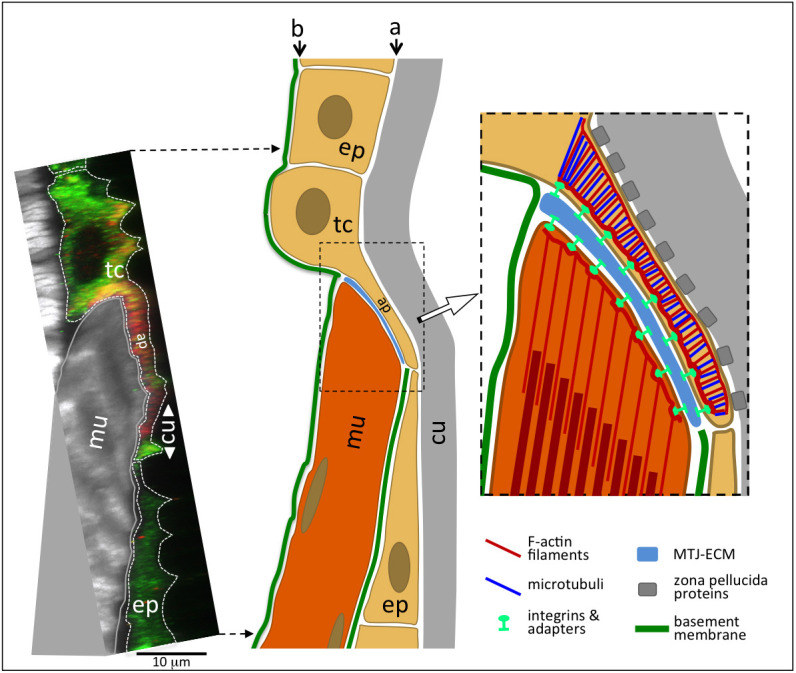
**Projection of side view and schematic drawing of direct MASs of a third-instar larva.** Shown on the left is a side view (confocal *YZ* projection and genotype as in Fig. 6A1) of a lateral transverse muscle (dorsal end) and its attached tendon cell. The tissues were stained with phalloidin (white), an anti-Zipper (Myosin II) antibody (green) and for F-actin-associated RFP (red) in the tendon cell. Corresponding schematic drawings are shown on the right. Arrows a and b indicate apical and basal sides, respectively. Abbreviations: ap, muscle attachment plate; cu, cuticle; ep, generic epithelial cell; MTJ-ECM, extracellular matrix of MTJ; mu, muscle; tc, tendon cell.

The tendon cells of direct MASs, which are our focus herein, form a specialized cellular subdomain positioned between the muscle tip and the cuticle (Fig. 1). This domain, which we term the attachment plate (Fig. 1), is quite thin (∼1–3 µm) and is oval shaped. The actual cell body (∼20–30 µm in diameter) including the nucleus is positioned to the side of this attachment plate (Prokop et al., 1998a; Alves-Silva et al., 2008) (Fig. 1). The apical–basal orientation of each tendon cell attachment plate mirrors that of its neighboring generic epithelial cells, with the apical side facing the cuticle and the basal side the respective muscle end. Apically, the attachment plate is anchored to the cuticle via extracellular matrix filaments composed of various zona pellucida domain (ZPD) proteins (Bökel et al., 2005; Chu and Hayashi, 2021). The muscle tip is anchored to the basal side of the tendon cell attachment plate via extracellular matrix containing Laminin [including the α1,2 chain Wing blister (Wb), the β chain LanB1 and the γ subunit LanB2], Perlecan (Trol), M-Spondin (Mspo), Thrombospondin (Tsp) with its partner Slowdown (Slow), Tiggrin (Tig), and the coagulation proteins Fondue (Fon) and Larval serum protein 1γ (Lsp1γ) (Fogerty et al., 1994; Yarnitzky and Volk, 1995; Martin et al., 1999; Friedrich et al., 2000; Chanana et al., 2007; Subramanian et al., 2007; Urbano et al., 2009; Wolfstetter and Holz, 2012; Green et al., 2016). Several of these proteins serve as ligands for integrins, which are imbedded in the basal membrane of the tendon cell attachment plate as αPS1 (Mew)–βPS (Mys) and αPS2 (If)–βPS heterodimers, and in the membranes at the muscle tips solely as αPS2–βPS heterodimers (Maartens and Brown, 2015). Loss of function of many of these MAS components leads to a weakening or rupturing of the muscle attachment, with the severity of the phenotype depending on the type of attachment (direct versus indirect), the site of action of the component, and molecular or functional redundancies. For example, in the prototypic case of null mutants for βPS, there is a complete rupture of muscle attachments once embryonic muscle contractions start towards the end of embryonic development, and all muscles ball up (Newman and Wright, 1981; Leptin et al., 1989). In mutants for components that are specific for tendon cells, only muscles attached directly are severely affected, because in indirect attachments the muscle–muscle attachments remain intact (Brown, 1994). Both in muscles and in tendon cells, the integrin heterodimers form hemiadherens junctions (Tepass and Hartenstein, 1994; Prokop et al., 1998a) with their cytoplasmic domains being linked to the cytoskeleton of the respective cell via dynamic integrin-associated protein complexes (reviewed in Maartens and Brown, 2015). Genetic loss of function or functional knock-downs within muscles cause various degrees of detachments and rounding of muscles, or detachment of F-actin filaments from the muscle membrane (reviewed in Maartens and Brown, 2015).

Within the tendon cell, the muscle contraction force is transmitted to the cuticle through a specialized, dense cytoskeletal array, also termed cytoskeletal belt, consisting of oriented microtubules and actin filaments that span the basal-to-apical axis of the attachment plate (Tepass and Hartenstein, 1994; Alves-Silva et al., 2008) (Fig. 1). The mechanical rigidity of this cytoskeletal array has been attributed to the ability of microtubules to resist strong tensile forces, combined with the elastic properties of F-actin networks that are strengthened by associated crosslinking factors and type II myosin (Alves-Silva et al., 2008).

To date, the best-characterized stabilizer of the cytoskeletal belt of larval tendon cells is the spectraplakin protein Short stop (Shot, also known as Kakapo) (Voelzmann et al., 2017). Shot is prominently expressed in tendon cells and is concentrated within the apical and basal portions of their cytoskeletal belts (Alves-Silva et al., 2008). The protein includes binding domains for actin, tubulin and the microtubule regulator EB1 (Röper et al., 2002). In the absence of functional Shot, the cytoskeletal belt of the tendon cell is destabilized, leading to significant stretching of the tendon cell and its cytoskeletal filaments. Consequently, the attached muscle tip retracts upon contraction (Gregory and Brown, 1998; Prokop et al., 1998b; Strumpf and Volk, 1998; Subramanian et al., 2003). Shot appears to mediate the assembly and anchorage of microtubule plus ends to the basal hemiadherens junctions within the tendon cell attachment plate. It is not currently clear whether actin binding is also involved in this function (Subramanian et al., 2003; Voelzmann et al., 2017). Additionally, Shot may promote the stabilization and bundling of microtubules (Bottenberg et al., 2009).

Herein we report on the identification of the presumed actin regulator Formin 3 (Form3) in a forward genetic screen as a major stabilizer of the cytoskeletal belt in tendon cells of larval direct muscle attachments. We show that in *form3* mutant larvae, the tendon cells of directly attached muscles become significantly stretched by the contracting muscles, which consequently snap back. This is accompanied by similar stretching, and in extreme cases detachment along the lateral sides, of F-actin fibrils and microtubule arrays within the tendon cells. Using an endogenously tagged Form3–HA, we demonstrate that Form3 is specifically expressed in larval tendon cells of both direct and indirect attachments, and that the protein is enriched in central apicobasal areas of the cytoskeletal belt of the attachment plate. Our findings provide important new insights into the regulatory activities involved in the assembly and stabilization of the specialized tendon cell cytoskeleton that allows these cells to resist the tension forces of muscle contractions.

## RESULTS

### New mutants *F958* and *C265* exhibit a specific phenotype in a subset of larval somatic muscles

**Fig. 2.**
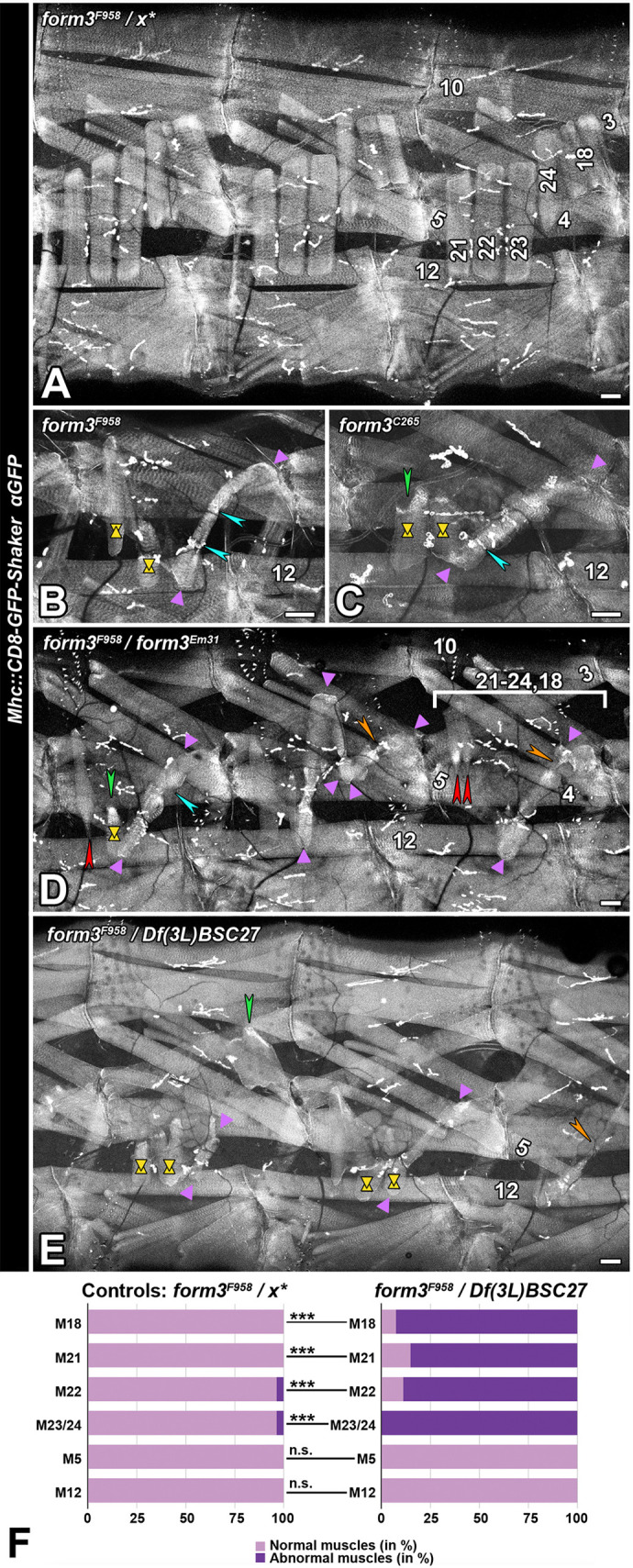
***formin 3* mutant third-instar larvae show detachments and aberrations in somatic transverse muscles.** Shown are third-instar larvae carrying *CD8-GFP-Shaker* in the background of different EMS mutations, as indicated. GFP signal is shown in white. Muscles of interest are numbered, including (for reference) some unaffected somatic muscles (muscles 3, 4, 5, 10 and 12). Anterior is left, dorsal is up. Scale bars: 50 µm. Images are representative of *N*=22 animals (A), *N*=1 (B), *N*=12 (C), *N*=2 (D), *N*=11 (E). (A) Control transheterozygote for *form3^F958^* and a complementing mutation from the screen show the wild-type pattern, including lateral transverse muscles 21–24, and muscle 18. (B) In a homozygous mutant *F958* larva, muscle 21 and muscle 22 (yellow shapes) are shortened ventrally and dorsally, respectively, and muscles 23, 24 and 18 seem to attach to each other to form a linear string of mini-muscles (between purple arrowheads; borders between muscles corresponding to presumed ectopic muscle-to-muscle attachments are labeled with turquoise arrowheads). (C) Homozygous mutant *C265* larva showing similar defects (labeled as in B), with muscle 21 additionally having a malformed dorsal tip with enhanced GFP signal (green arrowhead). (D) In larva transheterozygous for *F958* and the published *form3^Em31^*, all three hemisegments show defects in muscles 21–24 and muscle 18. In the most anterior hemisegment, muscle 21 has an abnormal spiky ventral tip (red arrowhead), muscle 22 has a shortened dorsal tip with enhanced GFP signal (green arrowhead; yellow shapes indicate muscle shortening), and muscles 22–24 and 18 are severely reduced, mispositioned, and apparently attached head-to-tail (turquoise arrowhead) to form a chain (between purple arrowheads). Analogous phenotypes in the other hemisegments are marked likewise. Thinly stretched attachments between muscles are marked by orange arrowheads. (E) The somatic muscle phenotype in larva transheterozygous for *form3^F958^* and *Df(3L)BSC27* resembles phenotypes shown above, with all lateral transverse muscles and muscle 18 being affected (labels as in B–D). (F) Quantification of the most commonly affected somatic muscles (M) compared to controls. Muscles 5 and 12 serve as non-affected examples. Control larvae: *n*=82 muscles, *N*=22 animals. *form3^F958^* over *Df(3L)BSC27* larvae: *n*=27 muscles, *N*=11 animals. ***P≤0.001; ns, not significant (Fisher's exact test).

In an ethyl methanesulfonate (EMS)-based screen for novel regulators of neuromuscular junction development (Aberle et al., 2002), several mutants with defects in the larval neuromuscular junctions were identified that were potentially an indirect result of altered somatic muscle morphologies. One of these, *F958*, is homozygous lethal in larval or pupal stages (although the lethality is increased by second site mutations on the chromosome, see below). Live imaging of the somatic muscles in an *F958* homozygous mutant larva shows strong defects in the pattern of lateral transverse muscles 21–24 (also known as LT1–LT4) and muscle 18 (DT1) compared to that in controls (Fig. 2B, compare with Fig. 2A). These muscles are shortened at their dorsal or ventral tips (Fig. 2B, yellow shapes). Some or all lateral transverse muscles and muscle 18 are reduced to abnormally shaped stumpy muscles and seem to ectopically attach to each other (Fig. 2B, turquoise arrowheads), resulting in a chain of short muscles (Fig. 2B, between purple arrowheads). Most oblique and all longitudinal muscles appear normal. The second mutation, *C265,* is semi-lethal. At 25°C, 7.5% of eclosing adults (of 241 flies analyzed) are homozygous for the mutant allele. These escapers show wings that are bent downwards and are not able to fly (data not shown, see also Fig. S1C). Homozygous *C265* larvae have defects in the larval somatic muscle pattern in the third-instar stage very similar to the homozygous *F958* phenotype (Fig. 2C). Complementation tests demonstrated that *F958* and *C265* are alleles of the same gene. Flies transheterozygous for *F958* and *C265* have a reduced eclosing rate (Table S1), all of them exhibit a bent-down wing phenotype, and transheterozygous larvae show a consistent expression of the somatic muscle phenotype (data not shown).

### *formin 3* is the mutated gene in alleles *F958* and *C265*

**Fig. 3.**
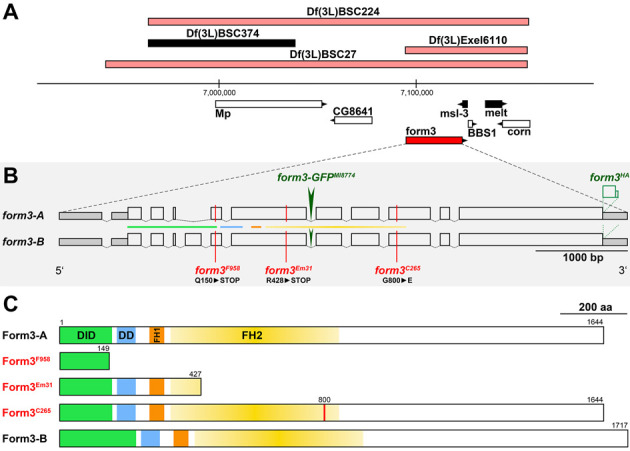
***formin 3* gene locus, alleles and their predicted protein products.** (A) *form3* gene locus. Deficiencies not complementing the wing phenotypes of alleles *form3^F958^* and *form3^C265^* are shown as light red bars. The complementing *Df(3L)BSC374* as well as the genes *msl-3* and *melt* (complementing when mutated) are shown as black bars. (B) Gene structure of *form3*. Two *form3* transcript variants, A and B, consist of 11 and 12 exons, respectively (open blocks represent coding sequence). An available Minos GFP protein trap (*MI08774*) is inserted in the sixth intron (dark green arrow). The sequence stretch containing two HA tags in our *form3^HA^* allele is added to the 3′ end of the ORF (green box). The positions and amino acids affected in the *form3* alleles *F958*, *C265* and *Em31* are indicated for isoform A. Colored lines refer to the encoded protein domains shown in C. Scale bar for exons: 1000 bp (introns not to scale). (C) Form3 protein structures of isoform A (1644 amino acids) and B (1717 amino acids). The Form3 N terminus harbors a diaphanous inhibitory domain (DID, green) and a dimerization domain (DD, blue) (Hegsted et al., 2017). Formin homology (FH) domains 1 and 2 (orange and yellow, respectively) were annotated in this work. Scale bar: 200 amino acids (aa).

In an effort to map the gene affected in *F958* and *C265*, we made use of the adult wing phenotype and screened the two alleles individually with a set of deficiencies uncovering the third chromosome. Both alleles showed a wing phenotype *in trans* to *Df(3L)BSC27*. Further screening of transheterozygous adults with shorter deficiencies and available mutants for several genes located in this region (Fig. 3A) revealed that only the mutant allele for *formin 3*, *form3^Em31^* (Tanaka et al., 2004) reproduces the wing phenotype *in trans* to our alleles (Fig. S1). We conclude that *F958* and *C265* are novel alleles of *form3,* a member of the formin family of cytoskeleton regulators.

When we compare the ratio of adults transheterozygous for each allele over a deficiency and the strength of the wing phenotypes between *form3^F958^*, *form3^C265^* and *form^Em31^,* it appears that *form3^F958^* and *form^Em31^* are stronger alleles than *form3^C265^* (Table S1).

### *form3* mutants show a consistent larval somatic muscle phenotype

The observation that *F958* is a novel allele of *form3* led us to examine the phenotype in larvae transheterozygous for the published *form3^Em31^* allele or a deficiency, which is expected to exclude any potential effects of second site mutations. Third-instar larvae that carry *form3^F958^ in trans* to *form3^Em31^* show a very similar phenotype in somatic muscles as homozygous *form3^F958^* animals, with all lateral transverse muscles 21–24 and muscle 18 being affected (Fig. 2D, compare with Fig. 2A). These muscles are shorter and misshapen, end at abnormal positions or form attachments with each other. Their tips are abnormally shaped, in most cases being elongated (Fig. 2D, red arrowheads) and displaying enhanced *CD8-GFP-Shaker* signals (Fig. 2D, green arrowhead). In several hemisegments we observe an apparent chain of short muscles that likely forms when aberrant lateral transverse muscles and muscle 18 ectopically attach to each other (Fig. 2D, between purple arrowheads; positions of abnormal attachment are labeled with turquoise arrowheads; stretched connections between muscles in such chains are indicated by orange arrowheads). In *form3^F958^/Df(3L)BSC27* larvae, lateral transverse muscles and muscle 18 show similar malformations in all analyzed hemisegments (Fig. 2E). In conclusion, *form3^F958^ in trans* to both *form3^Em31^* and a *form3* deficiency reproduces the somatic muscle phenotype we initially found in homozygous *form3^F958^*, and the observed phenotypes indicated the failure of normal muscle attachments in the absence of *form3* function.

To further support the observation that loss of *form3* function especially affects the group of lateral transverse muscles 21–24 and muscle 18, we compared the ratio of normal versus aberrant muscles in control and *form3^F958^/Df(3L)BSC27* third-instar larvae. Indeed, there is a very high abundance of abnormal muscles 18, 21, 22, and 23/24, in contrast to that for muscles 5 and 12 (Fig. 2F; Table S2). This allows the conclusion that in the lateral areas of the body wall loss of *form3* function specifically affects the lateral transverse muscles 21–24 and muscle 18. Late-stage larvae carrying *form3^C265^* over *form3^Em31^* or the *form3* deficiencies *Df(3L)Exel6110* and *Df(3L)BSC27* show a milder somatic muscle phenotype, with muscles 21, 22 and 18 often being unaffected or only mildly affected, again suggesting that *form3^C265^* is a weaker mutant allele of *form3* (data not shown).

### Exon sequencing characterizes *form^F958^* as a null allele and *form^C265^* as a hypomorphic allele of *formin 3*

To characterize the nature of the new *form3* alleles *F958* and *C265*, we re-analyzed the Form3 protein structure and mapped the EMS-generated mutations onto it (Fig. 3B,C). Form3 is a member of the highly conserved formin family of proteins, which are known regulators of filamentous actin and microtubules (Pruyne, 2016). The Formin homology 2 (FH2) domain is unique to and shared by all formins. Together with the formin homology 1 (FH1) domain, it provides formin proteins with the ability to nucleate and elongate actin filaments (Hegsted et al., 2017). The extent of the FH2 domain (Fig. 3C, yellow) was tentatively defined in this work since predictions of various protein analysis tools did not match exactly (Table S3).

The formin FH1 domain (Fig. 3C, orange) is defined as a region with one or more stretches of continuous proline residues, also called polyproline tracks, which are thought to form specific helices providing binding sites for profilin, a protein that binds actin monomers (Paul and Pollard, 2008). The Form3 FH1 domain lies N-terminally of the FH2 domain and has four repeats of seven successive prolines arranged as P_7_X_5_P_7_X_5_P_7_X_7_P_7_ (where X indicates any amino acid; Table S3).

Apart from the FH1 and FH2 domains, Hegsted, Yingling and Pruyne have also predicted two additional domains for *Drosophila* Form3: a diaphanous inhibitory domain (DID; Fig. 3C, green) at the N terminus, followed by a dimerization domain (DD; Fig. 3C, blue; Table S3) (Hegsted et al., 2017). The DID is thought to be important for protein regulation and/or subcellular localization. In other formins, it interacts with a C-terminal diaphanous autoregulatory domain (DAD), which results in a block of FH2 domain function (Valencia and Quinlan, 2021). In contrast to mammalian Form3 orthologs, *Drosophila* Form3 lacks a DAD domain.

Exon sequencing of *form3^F958^/w^1118^* DNA from adults showed a consistent double base read at genomic position 3L:7,117,988 (change of codon 150; Fig. 3B; Table S4). The mutation introduces a premature stop codon at the C-terminal end of the predicted DID and upstream of the predicted DD, FH1 and FH2 domains. Thus, the latter three domains are most likely lost in the mutant protein (Fig. 3C). In comparison, the premature stop in the *form3^Em31^* allele is positioned further downstream at the N-terminal end of the FH2 domain; all other conserved domains are still predicted to be present (Fig. 3C; Tanaka et al., 2004). Therefore, *form3^F958^* is potentially a stronger allele than *form3^Em31^*, and based on the absence of three of the four conserved protein domains, including both actin-regulating domains, it is very likely a null allele. Sequencing of *form3^C265^/+* DNA consistently produced a double base read at genomic position 3L:7,120,291, resulting in a conversion of glycine 800 to glutamic acid at the C-terminal end of our annotation of the FH2 domain (Fig. 3B,C; Table S4).

### Formin 3 is expressed in larval tendon cells

*Form3* mRNA expression in embryos has previously been detected in developing trachea, the anal pads and a few central nervous system (CNS) cells (Tanaka et al., 2004). As none of these tissues is thought to be connected with the above-described larval muscle phenotype, we analyzed the expression pattern of *form3* mRNA and protein in more detail, especially in larval stages.

**Fig. 4.**
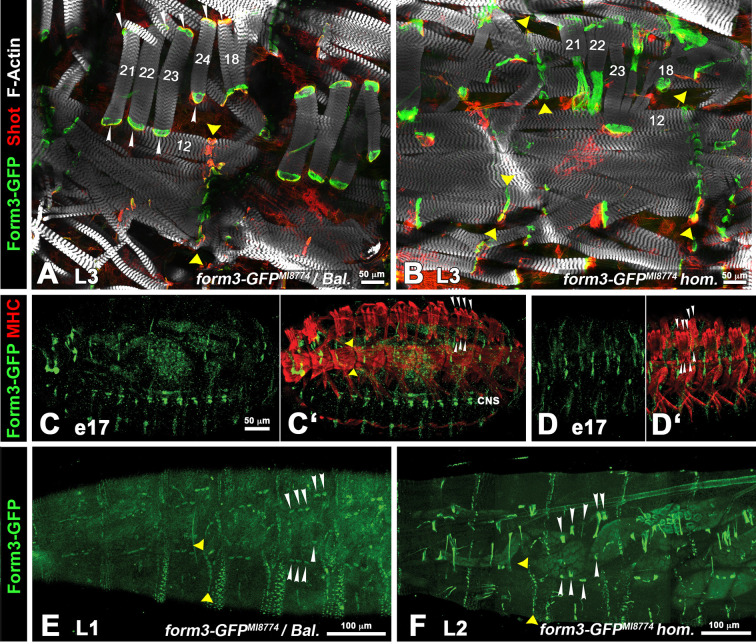
**Formin 3 is present in all larval tendon cells from first to third instar.** (A) Third-instar larva (L3) of *form3-GFP^MI08774^ in trans* to balancer (Bal.), stained with anti-GFP (green), anti-Shot (red) antibodies, and with phalloidin (white). Form3–GFP and Shot are co-expressed in tendon cells of both direct (muscle 18, muscles 21–24; white arrowheads) and indirect MTJs (yellow arrowheads). (B) In a homozygous (hom.) *form3-GFP^MI08774^* larva stained as in A, tendon cells of direct MTJs (muscle 18, muscles 21–23) are stretched and the attached muscles are shortened, unlike for indirect MTJs (yellow arrowheads). In A and B, muscles of interest are numbered. Scale bars: 50 µm. Images are representative of scans from four animals. (C–D′) Stage 17 *form3-GFP^MI08774^* embryos (e17) stained with anti-GFP (green) and anti-MHC (myosin heavy chain; red) antibodies. Form3–GFP is expressed in neurons of the CNS but not in tendon cells, including those of lateral transverse muscles (white arrowheads). Scale bar: 50 µm (C–D′). Images are representative of scans of five embryos. (E) In a first-instar larva (L1) of *form3-GFP^MI08774^ in trans* to balancer, live imaged Form3–GFP is expressed in tendon cells of lateral transverse muscles and muscle 18 (white arrowheads) and in tendon cells along the segment borders (yellow arrowheads). (F) In a homozygous second-instar larva (L2) of *form3-GFP^MI08774^*, live imaged Form3–GFP continues to be expressed in both types of (variously elongated) tendon cells marked as in E. Scale bars: 100 µm (E,F). Images are representative of three scanned larvae and >10 visually inspected larvae. In all panels, anterior is to the left and dorsal is up. The color codes for each channel are provided on the left of each row.

An available GFP protein trap insertion in *form3, form3-GFP^MI08774^* (hereafter called *form3-GFP)* is semi-lethal. The insertion site is in the region encoding the FH2 domain (Fig. 3B, dark green arrow; Tables S3, S4). To avoid any detrimental effects of the GFP knock-in on Form3 expression or function we first analyzed the fusion protein in a heterozygous condition. In late third-instar larvae, Form3–GFP is expressed in all larval tendon cells, which can be identified by the expression of the spectraplakin protein Shot (Fig. 4A) (Voelzmann et al., 2017).

In embryos, neither *form3* mRNA nor Form3–GFP protein (even at late stage 17) can be detected in tendon cells (Fig. 4C–D′; Fig. S2 and data not shown). Instead, embryonic Form3–GFP expression includes tissues previously known for *form3* mRNA expression, particularly trachea, neurons of the CNS and the anal plate, and in addition tissues with previously unreported *form3* mRNA expression, particularly pericardial cells and the fat body (Fig. 4C–D′; Fig. S2). Notably, with live imaging we were able to detect Form3–GFP in tendon cells in first-instar larvae (Fig. 4E), as well as in second-instar larvae (Fig. 4F). These data, together with those using an HA-tagged version of Form3 (see below), show that Form3 expression in tendon cells initiates at the transition from embryonic stage 17 to the larval first instar.

Third-instar larvae homozygous for *form3-GFP* show a muscle attachment phenotype that is qualitatively similar to that observed with the *form3* EMS alleles. Many tendon cells marked by Form3–GFP and Shot have an abnormal morphology as they are stretched and thin; conversely, the lateral transverse somatic muscles and muscle 18 attached to them are significantly shortened (Fig. 4B, compare with Fig. 4A). By contrast, in late-stage embryos transheterozygous for two EMS alleles, *form3^F958^*/f*orm3^Em31^*, all muscles appear completely normal (Fig. S3A–A″, compare with Fig. S3B–B″). Importantly, however, live imaging with CD8–GFP–Shaker revealed that first-instar *form3^F958^*/*form3^Em31^* larvae display strongly shortened and misshapen lateral transverse muscles and muscle 18 (Fig. S3C,I, compare with Fig. S3F,L). Because analogous phenotypes are detected in second- and third-instar larvae (shown for tendon cells in Fig. 4F and for muscles in Fig. S3D,E,J,K, compare with Fig. S3G,H,M,N) we aimed to measure the temporal progression of the shortened muscle phenotype of lateral transverse muscles and muscle 18 through the larval stages quantitatively. As seen in Fig. S4, the bulk of muscle shortenings already occur in first-instar larvae although, particularly for muscle 18, there appears some additional shortening relative to the controls during the second and third instar.

Taken together, the postembryonic initiation of Form3 expression and function suggests that its role is restricted to stabilizing larval tendon cells starting from the first instar, without being involved in the formation of embryonic muscle–tendon attachments.

### Loss of *formin 3* results in muscle attachment failure at direct MTJs

**Fig. 5.**
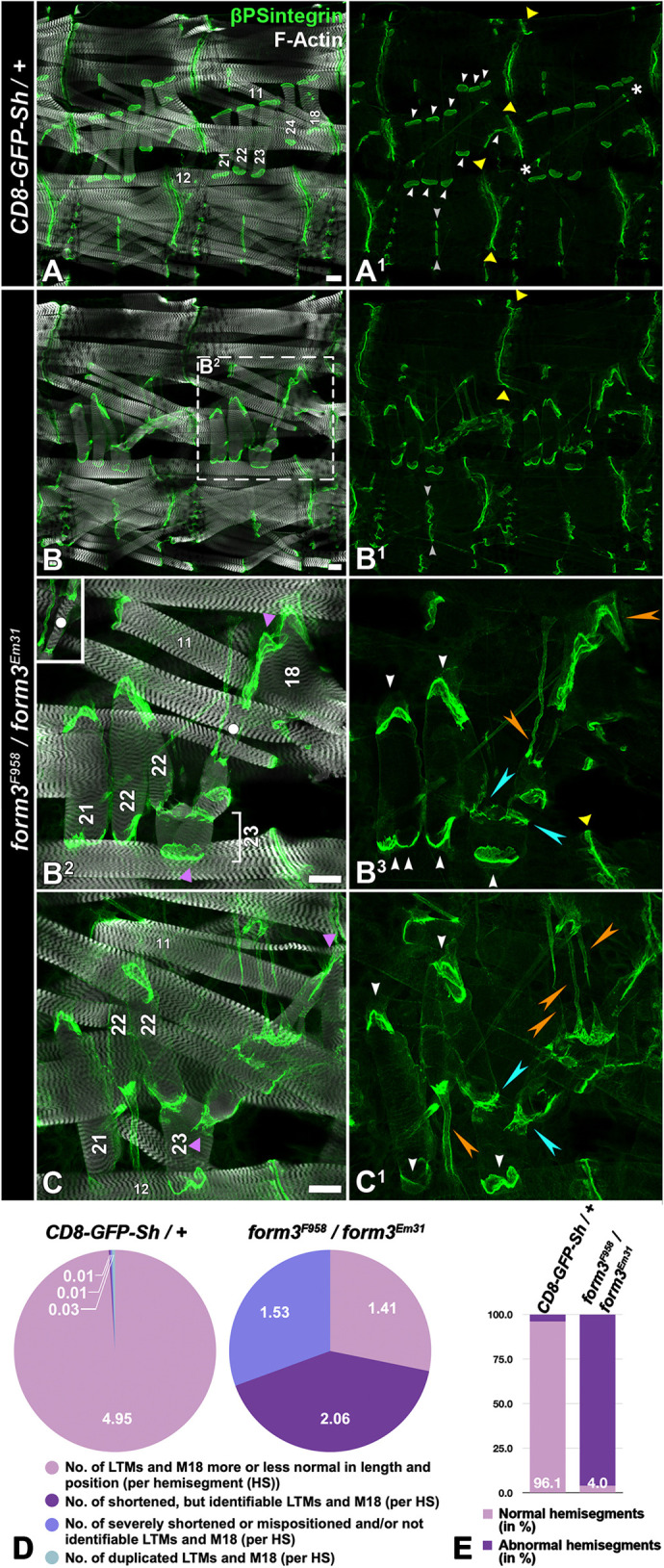
**Loss of *formin 3* results in loss of tendon cell integrity and muscle retraction at direct MTJs.** Shown are third-instar larvae stained with phalloidin (F-actin; grey) for muscles and anti-βPS integrin antibodies (green), which label tendon cells and the chordotonal organ (between white asterisks in A1). Muscles 11, 12, 21–24 and 18 are numbered for reference. Anterior is left, dorsal is up. Scale bars: 50 µm. (A,A1) In controls of the indicated genotype, lateral muscles 21–24 and muscle 18 are attached at direct MTJs (white arrowheads), similar to the more ventral intrasegmentally attached muscles (between grey arrowheads). At the segment border, muscles are indirectly attached (between yellow arrowheads). (B–C1) Mutant larvae of the indicated genotype show abnormalities in almost all lateral muscles 21–24 and 18. Corresponding tendon cells are typically more stretched than crescent-shaped (compare A1 with B3 and C1, white arrowheads). Muscles are often forked at one end (e.g. muscle 22 in B2,C). Muscles frequently are shortened and either attach to a strongly elongated tendon cell (orange arrowheads) or ectopically to an opposing muscle tip (turquoise arrowheads). Muscles 24 and 18 are often not identifiable due to the presence of short muscle remnants attached to each other forming a string of mini-muscles (between purple arrowheads; inset in B2 shows mini-muscle marked by dot in a different *Z* plane). Yellow arrowheads, indirect MTJs. (D) Quantification of muscle morphology of muscles 21–24 (lateral transverse muscles, LTMs) and muscle 18 (M18) (five muscles per hemisegment) in control and mutant backgrounds (*n*=80 control and 100 mutant hemisegments analyzed in *N*=12 larvae per genotype) as shown in A–C1. (E) Ratio of hemisegments with any of the muscle abnormalities listed in D in controls (*n*=80 hemisegments, *N*=12 animals) versus in *form3* loss-of-function mutant (*n*=100 hemisegments, *N*=12 animals).

To analyze the larval *form3* loss-of-function phenotype in more detail, we used antibodies against βPS integrin, which localizes to the opposed membranes of both tendon cells and muscle tips. Third-instar *form3^F958^*/*form3^Em31^* larvae show abnormalities in several muscles that coincide with abnormally shaped tendon cells at their attachment sites (Fig. 5B–B3 and Fig. 5C,C1, compare to control in Fig. 5A,A1). Again, the phenotype is most prominent in the group of lateral transverse muscles 21–24 and adjacent muscle 18. All muscles in this group are shortened, malformed and wrongly oriented, and their tendon cells are extended to various degrees (e.g. Fig. 5B2,B3,C,C1, orange arrowheads). Muscles seem to abnormally form attachments to each other. As a result, muscles of this group often appear to form a chain of shortened muscles as described above for *form3^F958^* and *form3^C265^* mutant backgrounds. At the interfaces of these abnormal muscle-to-muscle attachments we observe βPS integrin accumulation (Fig. 5B2,B3,C,C1, turquoise arrowheads, and similarly in hemizygous *form3^Em31^* larvae, Fig. S5A,A3,A4, arrows). Low and fragmented signals of the tendon cell marker β1-Tubulin (βTub56D) are also present at these abnormal muscle–muscle interfaces in *form3* mutant larvae (Fig. S5B). One end of these muscle chains is usually still connected to the cuticle via an elongated tendon cell (Fig. S5A4). We speculate that these peculiar arrangements are the result of secondary processes occurring after the complete rupture of some elongated tendon cells, when the presence of adhesive material at the membranes of muscle ends – including integrins, extracellular matrix and fragmented tendon cell material – might promote ectopic attachments of released muscle ends. However, due to the impossibility of live imaging in the moving larvae, the exact processes establishing these abnormal muscle configurations upon muscle detachments are very difficult to ascertain.

**Fig. 6.**
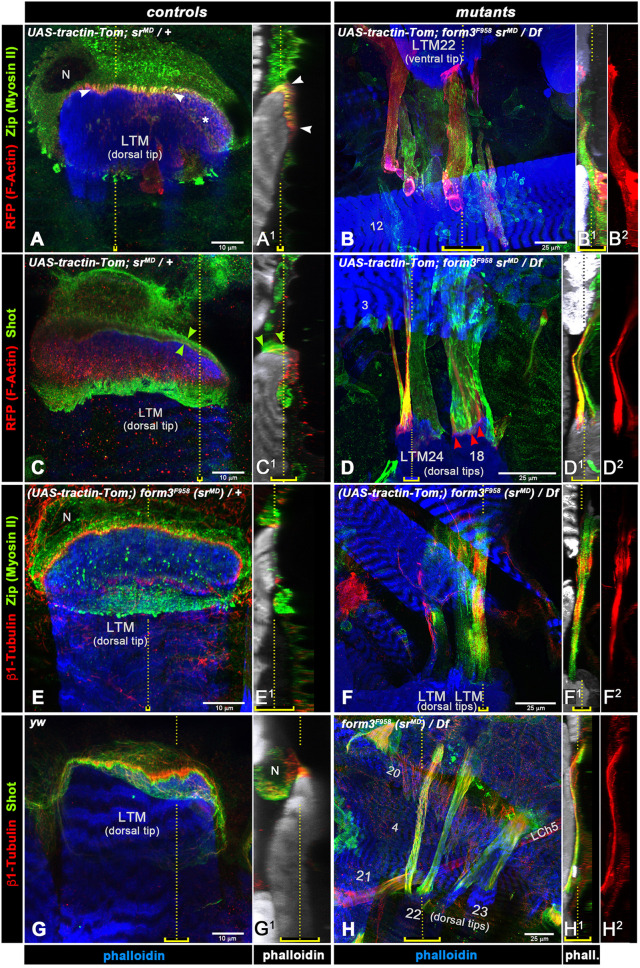
**Effects of *form3* mutations on the distributions of known cytoskeletal tendon cell markers in third-instar larvae.** Shown are high-magnification views of tendon cells attached to their respective lateral transverse muscles (numbered if identified). For the controls, single tendon cells are shown (scale bars: 10 µm), whereas for the mutants, two to three tendon cells are shown at slightly lower magnification to accommodate their dramatically increased lengths (scale bars: 25 µm). Dorsal ends of LTMs are shown except for B–B2, which show ventral muscle ends. For each panel, the left-hand image shows a top-down view of tendon cells with their attached muscles (phalloidin in blue; *XY* scans merged in the *Z* dimension), and images on the right show side views of the same respective muscles (phalloidin in white; *YZ* re-slices merged in the *X* dimension; epidermis oriented to the right). Yellow dotted lines in the top-down views indicate the positions used for generating the corresponding *YZ* images of the side views, with the yellow brackets indicating the extent of the *X*-axis projection in the side views. Likewise, yellow dotted lines and brackets in the side-views indicate the positions and extents of *Z*-axis projections used for the images in the corresponding *XY* top-down views. Controls had either *UAS* and *GAL4* chromosomes over+(A,A1,C,C1), *form3^F958^* on *GAL4* driver (*sr^MD710^*) chromosome over+(E,E1), or were *yw* (G,G1). Mutants carried the *form3^F958^* point mutant allele in *trans* to *Df(3L)Exel6110*, together with the *UAS* and *GAL4* insertions as indicated. Genetic elements shown in brackets were present but not functionally relevant for the data shown. The antibody color codes are shown on the left and for phalloidin on the bottom. (A,A1) In control, F-actin (visualized via *sr>tractin-Tomato*) and Zipper (Zip; Myosin II) are colocalized prominently in cables (arrowheads; asterisk marks transverse sections) within the narrow cytoskeletal belt of the tendon cell between the muscle end and cuticle. Zip is also present in the tendon cell body except for its nucleus. (B–B2) In *form3^F958^* mutants, F-actin and Zip are distributed throughout the elongated tendon cells. (C,C1) In control, F-actin and Shot partially colocalize in cables within the cytoskeletal belt of the tendon cell. Green arrowheads denote stronger Shot signals at apical and basal positions of the MTJ. Shot signals are much weaker in central areas of the attachment. (D–D2) In *form3^F958^* mutants, both F-actin and Shot are distributed throughout the extremely elongated tendon cells and only partially colocalized, as F-actin arrays are separated laterally (red arrowheads). (E,E1) In control, β1-Tubulin is present in the cytoskeletal belt as well as in the tendon cell body. As for Shot, β1-Tubulin signals are very weak in central areas of the attachment plate and largely distinct from Zip. (F–F2) In *form3^F958^* mutants, long β1-Tubulin-containing structures are present along the elongated tendon cells, albeit with interruptions, including near a muscle tip (F1,F2). Extended Zip is distributed as in B. (G,G1) In control, β1-Tubulin and Shot are partially colocalized but mainly adjacent to each other within the cytoskeletal belt of the tendon cell. (H–H2) In *form3^F958^* mutants, both β1-Tubulin and Shot extend all along the elongated tendon cells, in this example with lateral splits but without any major interruptions. Abbreviations: LCh5, lateral chordotonal organ 5; LTM, lateral transverse muscle; N, tendon cell nucleus. Images are representative of the total scans indicted in the Materials and Methods (‘Microscopy’ section).

Remaining shortened lateral transverse muscles that are not connected to each other, as well as other shortened muscles that utilize direct attachments to tendon cells and the cuticle – such as muscle 11 (dorsal ends), muscle 27 (ventral ends) and the ventral muscles 16 and 17 (ventral ends) – show abnormal pointy or frayed tips with high F-actin densities, which is accompanied by attached βPS integrin-positive tendon cells that are strongly elongated (Fig. 5B–C1; Fig. S5A–A2).

Quantifications of lateral transverse muscle phenotypes (Fig. 5D, individual categories; Fig. 5E, numbers of affected hemisegments) show a very high penetrance. In addition, severely stretched tendon cells and ectopic head-to-tail attachments of muscles 21–24 or muscle 18 were quantified in control versus mutant larvae. Only clearly identifiable muscles were analyzed. Among these five analyzed muscles in a *form3^F958^*/*form3^Em31^* loss-of-function background, a mean of 1.72 tendon cells per hemisegment (*n*=100 hemisegments) are stretched severely, and a mean of 1.32 ectopic head-to-tail muscle attachments per hemisegment were present. Controls show a mean of 0.01 elongated tendon cells per hemisegment (*n*=80) and no ectopic head-to-tail muscle attachments.

In summary, where muscles (particularly lateral transverse muscles, muscle 11 and ventral oblique muscles) are shortened and show abnormal pointy tips, the attached tendon cells are stretched correspondingly and exhibit a tube-like morphology (see additional evidence below). The stretched tendon cells in a *form3* loss-of-function background appear to result from failed tendon cell integrity, leading to an elongation of the tendon cells and a corresponding shortening of the muscles upon their sustained contraction during larval stages. Notably, only muscle tips that are attached intrasegmentally and thus are part of direct MTJs (as opposed to indirect MTJs at the segment borders) are affected in this fashion. Therefore, Form3 appears vital for stabilizing the mechanical integrity chiefly of the tendon cells of direct MTJs.

In the adult thorax, the indirect flight muscles are also attached directly, although their tendon cells exhibit a different morphology as compared to those of the larval direct MTJs. Form3, as detected via Form3–GFP in the above-described gene trap line, is also expressed in all thoracic tendon cells (Fig. S6A). In adult *form3^F958^* hemizygous escapers, we observe that both the dorsoventral (Fig. S6C, compare with Fig. S6B) and the dorsal longitudinal (Fig. S6E,F, compare with Fig. S6D) indirect flight muscles are retracted to various degrees from the thoracic cuticle and their respective tendon cells are extended. Thus, Form3 continues to contribute to the stability of the tendon cell cytoskeleton in the adult stages.

### Form3 is required for tensile strength of the cytoskeletal belt within tendon cells

Next, we examined the subcellular consequences of *form3* mutations at the attachment sites at a higher resolution. Previous studies have demonstrated that the rigid connection between muscles and cuticle is mediated by a thin cellular plate formed by specialized portions of each tendon cell. This structure contains a compact array of actomyosin filaments and microtubules that are anchored at the apical and basal sides of the cellular plate, which has been termed the ‘cytoskeletal belt’ of the MTJs (Alves-Silva et al., 2008) (Fig. 1). In our experiments, we detected F-actin by driving tendon cell-specific expression of *UAS-tractin-Tomato* with *sr^MD710^-GAL4* (*stripe*>*tractin-Tomato*), followed by anti-RFP and phalloidin staining. The spectraplakin Shot as well as Zipper (Zip; also known as Myosin II) and β1-Tubulin, which are also highly abundant in tendon cells, were detected with antibodies against these proteins (Buttgereit et al., 1991; Jordan and Karess, 1997; Strumpf and Volk, 1998).

In controls with one functional copy of *form3*, F-actin and Zip colocalize in the compact cable-like structures of the cytoskeletal belt of the MTJ forming the direct attachment of a lateral transverse muscle to the cuticle [Fig. 6A, top-down view (i.e. *Z*-stack projections of several *XY* scans); Fig. 6A1, side view of the same muscle, see arrowheads]. Unlike F-actin, Zip is also present, at slightly lower levels, in the tendon cell body, except for the nucleus (Fig. 6A,A1). In top-down views, the F-actin- and myosin-decorated fibrils are evident as dots (Fig. 6A, asterisk), whereas at the edge of the attachment plate and in side views they are seen as short streaks (Fig. 6A,A1, arrowheads). The outlines of the tendon cell and muscle shown in Fig. 6A,A1 are marked in Fig. S7, where single-channel views are shown for additional clarity. In contrast to the control, F-actin filaments at the corresponding MTJ of a *form3^F958^*/*Df(3L)Exel6110* mutant larva are present throughout the length of the elongated cytoskeletal belt and appear to be split laterally into two or three strands (Fig. 6B–B2 and Fig. S7B,B′; compare with Fig. 6A and Fig. S7A,A′; note the different magnifications of controls versus mutants). Zip distribution is more uneven as compared to F-actin (Fig. 6B,B1). Areas showing Zip but no F-actin include the tendon cell body, which is also stretched albeit to a lesser extent as compared to the attachment plate, as it is already much wider in the normal situation (Fig. 6B; see Fig. S7B–C for cellular outlines, single channels and the particular location of these structures within the segment).

In the control, Shot partially overlaps with F-actin within the myotendinous cytoskeletal belt and is also seen in the tendon cell body (Fig. 6C,C­1). Increased Shot levels are present at the apical and basal sides of the belt (Fig. 6C, arrowheads; see also Alves-Silva et al., 2008). Shot staining within the attachment plate is strongest at the edge(s) nearest to the cell body, across from the distal muscle tip, and much weaker staining is seen in the middle of the F-actin-positive plate (Fig. 6C,C1) (it is currently not known whether these unequal signals across the plate reflect reduced antibody accessibility in the compact center of the plate, as discussed in Alves-Silva et al., 2008, or the genuine protein distribution). In the *form3* mutant with extremely elongated attachment structures shown in Fig. 6D–D2 (see Fig. S7D for neighboring muscle context), the elongated F-actin fibrils in part have separated laterally (Fig. 6D, arrowheads) and discontinuities along their length are seen. There is no strict colocalization of F-actin with Shot, as some F-actin-positive areas lack Shot signals and some low-F-actin sites show high levels of Shot (Fig. 6D,D1). Similar extreme elongations of F-actin fibrils with occasional thinning and even ruptures along their lengths can be seen with the direct attachments of ventral oblique muscles in *form3* mutant larvae (Fig. S8).

β1-Tubulin is present in short tubules in the attachment plate of control larvae, although the signals are restricted to peripheral areas of the plate, presumably again due to limited antibody accessibility in central areas (Fig. 6E,E1; see also Alves-Silva et al., 2008). Zip signals are seen in short apicobasal fibrils, as in Fig. 6A,A1, and partially overlap with β1-Tubulin signals where these are present. In the *form3* mutant shown in Fig. 6F–F2, the microtubule arrays are vastly elongated along with the attachment plate and body of the tendon cell, and several interruptions are present along the length. Zip signals are seen mostly at the periphery of the microtubule arrays and in areas devoid of them (Fig. 6F,F1; Fig. S7E). In double stainings for β1-Tubulin and Shot, the two signals largely overlap within the MTJ both in the control and in the *form3* mutant, and in the mutant they are spread throughout the extremely extended junction (Fig. 6H–H2, compare with Fig. 6G,G1; see Fig. S7F for the context of surrounding muscles). In this example, lateral splits but no major interruptions along the length of the microtubule arrays are present.

To test the possibility that other members of the formin family contribute to the stability of the tendon cell cytoskeleton we performed RNAi knock-downs for *DAAM*, *Fhos*, *Frl*, *mwh*, and *form3* in tendon cells. As Form3 and Dia orthologs from humans and *Caenorhabditis elegans* have been reported to inhibit each other (Sun et al., 2013; Shaye and Greenwald, 2016), Dia was overexpressed as a wild-type GFP fusion protein and as a constitutively active version in tendon cells. As shown in Fig. S9, only *form3* knock-down produced the archetypic muscle attachment phenotype described for the mutants, suggesting that these other *Drosophila* formins do not have essential roles in this context.

### Form3 prominently localizes to the cytoskeletal belt of the MTJs within tendon cells

The crucial role of Form3 in maintaining the integrity of direct muscle attachments led us to examine the precise subcellular localization of Form3 within tendon cells and correlate it with components of the cytoskeletal belt of MTJs. As Form3–GFP represents an in-frame insertion of GFP within the conserved FH2 domain and *form3-GFP* mutants display a muscle attachment phenotype, the localization of Form3–GFP might not accurately reflect the localization of fully functional Form3. Therefore we generated a *form3* allele with HA tag sequences attached to the 3′ end of the open reading frame (ORF) at its native locus via CRISPR/Cas9 (see Materials and Methods). The absence of a muscle attachment phenotype in homozygous larvae for this allele confirmed that the short HA tag at the C terminus of the expressed Form3–HA does not significantly interfere with its function and, presumably, localization. Embryonic expression of Form3–HA is observed in the same tissues as seen for Form3–GFP (Fig. S2). Minor differences exist with regard to protein localization, such as the presence of Form3–HA in axons of CNS neurons, whereas Form3–GFP appears restricted to the cell bodies (Fig. S2N,Q). The general absence of Form3 expression in embryonic tendon cells, as seen with Form3–GFP, was confirmed with Form3–HA (Fig. S2P and data not shown). We only found a single stage 17 embryo with Form3–HA tendon cell expression, which likely happened to have a permeable cuticle for the antibodies (Fig. S2R), thus further indicating that Form3 expression initiates at the transition between late embryonic and first-instar larval stages.

**Fig. 7.**
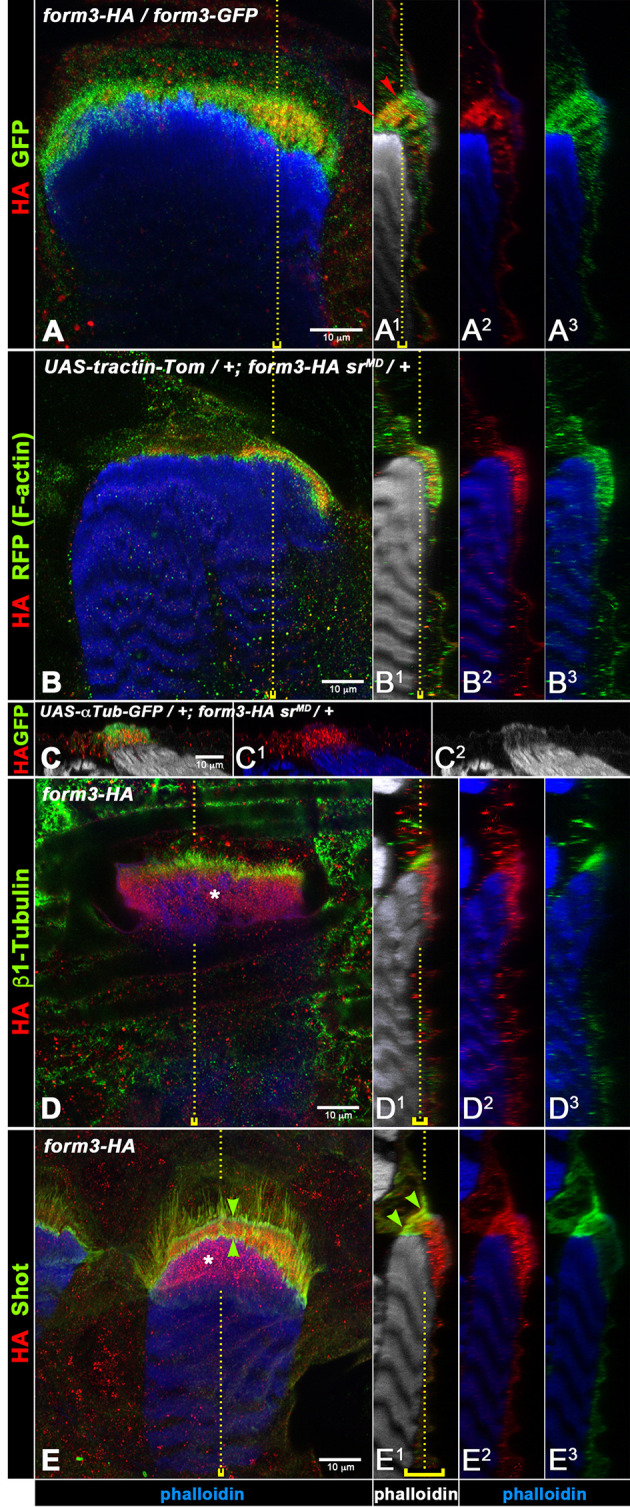
**Localization of Form3 within the cytoskeletal belts of tendon cells at MTJs.** Shown are the dorsal ends of lateral transverse muscles with their respective tendon cells from third-instar larval filets expressing endogenous Form3 fused to HA-tag sequences at the C terminus, together with other markers (as indicated on the left for each panel). Genotypes are shown within each row. Phalloidin staining is shown in blue or white (as indicated below the images). Scale bars: 10 µm. The left-hand column shows top-down views (*XY* scans merged in the *Z* dimension) and the right-hand columns show side views of the same muscles (*YZ* re-slices merged in the *X* dimension) in three different channel combinations. Yellow dotted lines and brackets are shown as explained in the legend of Fig. 6. The side views are oriented with the cuticle to the right except for C–C2, where the cuticle is towards the top. (A–A3) Simultaneous detection of Form3–HA and Form3–GFP in a larva carrying the *form3^HA^* and *form3-GFP^MI08774^* alleles *in trans*. Both tagged versions of Form3 colocalize in cables in the center of the cytoskeletal belts of the attachments, but Form3–GFP is also present in basal areas close to the muscle end and apically near the cuticle, and more weakly also in the tendon cell body, where Form3–HA levels are very low or absent (red arrowheads mark outer limits of the Form3–HA domain). (B–B3) Form3–HA overlaps with F-actin as visualized via *sr>tractin-Tomato* (using an anti-RFP antibody) in the cytoskeletal belt within the tendon cell, although Form3–HA is more concentrated in central areas. (C–C2) Form3–HA and αTubulin (as visualized via *sr>αTub-GFP*) overlap in the cytoskeletal belt, although Form3–HA (C1) is found more strongly in its center, whereas αTubulin (C) and F-actin (C2) are stronger particularly near its apical end. (D–D3) Form3–HA and β1-Tubulin colocalize in the cytoskeletal belt of the tendon cell, although β1-Tubulin staining is largely restricted to the area next to the cell body. (E–E3) Form3–HA and Shot colocalize in the cytoskeletal belt of the tendon cell, but Shot staining is largely restricted to the area of this belt next to the cell body, is stronger apically and basally within this belt, and is present in fibers extending into the cell body (arrowheads indicate apical and basal areas with high levels of Shot). Asterisks in D and E indicate cross-sectioned Form3–HA cables. Images shown are representative of the total scans indicted in the Materials and Methods (‘Microscopy’ section).

In third-instar larvae, we first compared the subcellular localization of Form3–HA and Form3–GFP in MTJs of lateral transverse muscles of *form3^HA^*/*form3-GFP* transheterozygotes. As shown in Fig. 7A–A3 (images arranged and processed as in Fig. 6), Form3–HA is detected in apicobasal ‘streaks’ within the MTJ but is absent in the apical-most areas marked by phalloidin and in basal-most areas close to the muscle (Fig. 7A2). Form3–GFP overlaps with Form3–HA in these fibrils, but unlike Form3–HA, which is enriched in central areas of the attachment plate (Fig. 7A1, arrowheads), Form3–GFP is also present in the apical and basal areas. In addition, Form3–GFP distribution in the MTJ is more even and shows a clearer presence in the tendon cell body as compared to Form3–HA (Fig. 7A,A1,A3).

As formins are known for their roles as F-actin regulators, it was pertinent to examine the localization of Form3–HA relative to F-actin in MTJs. Fig. 7B–B3 shows that the Form3–HA ‘streaks’ in some cases overlap with the F-actin fibrils and in other cases are positioned in close vicinity to them. Like in Fig. 7A–A2, Form3–HA is limited largely to the middle region of the cytoskeletal belt, whereas the F-actin arrays span its entire apical–basal extent (Fig. 7B–B3; see also Fig. 7C1,C2). Similarly, Form3–HA overlaps with the microtubule arrays in the MTJs, as is evident in double stainings for αTubulin–GFP (expressed via *stripe>αTub-GFP*; Fig. 7C–C2) and for β1-Tubulin (Fig. 7D–D3). Again, the tubulin signals extend along the entire apicobasal extent of the MTJs, whereas Form3–HA is largely limited to the central areas. Colocalization of Form3–HA and Shot is also seen, although as already noted, highest levels of Shot are present in both apical and basal areas of the MTJ (Fig. 7E–E3, arrowheads), whereas Form3–HA displays the opposite quantitative distribution along this axis. Taken together, these data indicate that Form3 is largely restricted to, and aligned with, mid-fiber regions of F-actin filaments and microtubules.

## DISCUSSION

### Formin 3 – a new player in the mechanical reinforcement of tendon cells

In this study, we identified Form3 as a crucial player enabling larval tendon cells, specifically those at direct muscle attachments, to withstand the strong tensile forces generated by muscle contractions. Form3 acts within the muscle attachment plates of these tendon cells by reinforcing the specialized F-actin and microtubule arrays spanning these structures between their basal and apical hemiadherens junctions. These cytoskeletal belts transmit the forces between muscle and cuticle (Alves-Silva et al., 2008). Because *form3* mutants do not exhibit any defects in embryonic muscles or tendon cells, and because Form3 is not expressed in embryonic tendon cells, we can exclude the possibility that Form3 plays a role in the formation of muscle attachments and functional differentiation of tendon cells. It is plausible that muscle forces increase significantly upon onset of contraction movements at the very end of embryonic development, and that vigorous movements of the much larger muscles during larval instars necessitate additional cytoskeletal strengthening. A similar rationale may explain the absence of an overt muscle phenotype in larval indirect muscle attachments, where forces primarily act between the opposing muscle ends, with less force expected to act on the perpendicular connections to the tendon cells between the muscle ends and the cuticle. The relatively mild phenotype observed in tendon cells of the indirect flight muscles, where Form3 is also expressed, could be attributed to activities that act partially redundantly with Form3 there, perhaps including Filamin (also known as Cheerio) (Manieu et al., 2018). However, in larvae starting from first instar, the lateral transverse muscles, which attach directly at both their dorsal and their ventral ends, are particularly affected by the loss of Form3 activity. Typically, attachment at one end (either dorsal or ventral) fails, whereas the other end is not or only mildly affected. This is likely due to the relief of the contractile force once the attachment on the other side gives way. The affected tendon cells can be extensively stretched, in most cases without breaking. However, we regularly observe peculiar chains or Y-shaped arrangements of shortened muscles at the normal positions of the lateral transverse musculature. We propose that in these cases, the tendon cells have snapped and the muscle ends, by virtue of their adhesive ends containing a specialized extracellular matrix and some residual tendon cell material, have engaged in connections to ends of their snapped-off neighbors, somewhat akin to the normal situation at indirect muscle attachments.

### Possible molecular and cellular functions of Form3 in the F-actin and microtubule arrays

What could be the molecular and cellular role of Form3 in reinforcing the cytoskeletal belts of tendon cells? The canonical function of formins is to polymerize and elongate actin filaments at their barbed ends in a linear fashion (Valencia and Quinlan, 2021). This process involves encircling the barbed end with the dimerized FH2 domains of formin homodimers, recruiting profilin-bound actin to the FH1 domains and advancing along the elongating barbed end to continue its elongation (Valencia and Quinlan, 2021). It is reasonable to assume that Form3 contributes to the elongation of actin filaments in tendon cells in this fashion. This role could be relevant in dynamic processes such as the elongation of the cytoskeletal belts as a response to strong muscle contraction, potentially explaining the observed variable thickness of the cytoskeletal belts at MTJs of the same type of muscle in individual fixed wild-type larvae. Form3 might also engage in actin filament elongation upon the lateral addition of new actin filaments during the extensive growth of the attachment plates during larval stages. However, it has been shown that the cytoskeletal belts of larval tendon cells form compact arrays with rather limited dynamic properties that are very different from those of stress fibers. For example, although cytochalasin treatment has been found to destroy most actin filaments in tendon cell bodies, those in cytoskeletal belts of the attachment plate remain largely intact (Alves-Silva et al., 2008). Likewise, the same authors have reported that there are no immediate effects on the cytoskeletal belts upon experimental muscle detachment, also indicating their rigidity and low dynamism (Alves-Silva et al., 2008). In *form3* mutants, we observe that the F-actin fibrils elongate along with the tendon cell rather than shorten, remaining continuous along their length except in extreme cases. This phenotype suggests the presence of additional F-actin elongation activities in the tendon cells, and Form3 might even restrict their actin polymerization activities.

Previous findings on other formins, particularly mammalian orthologs of Form3 such as INF1 (FHDC1) and INF2, might provide further insight into the role of Form3 in tendon cells. INF2 has been reported to be one of the slowest elongating formins (Gurel et al., 2015; Palmer et al., 2024). Importantly, unlike other formins such as mDia (DIAPH1), INF2 has a preference for mid-filament binding over barbed-end binding, which involves opening and re-closing its dimeric FH2 ring around the filament (Gurel et al., 2014; Palmer et al., 2024). This preference might be relevant for the understanding of Form3 function in tendon cells.

Firstly, internal binding of INF2 is associated with a severing activity of actin filaments by INF2, generating multiple short filaments that are either depolymerized or elongated, depending on the monomeric actin equilibrium and perhaps on regulatory activities in the cell (Chhabra and Higgs, 2006; Gurel et al., 2014, 2015). If Form3 shares this F-actin-severing activity with INF2, it could promote rapid actin filament amplification during the extensive growth of the tendon cell attachment plates in larvae.

Secondly, INF2, its paralog INF1 and some other formins have been shown to bind and stabilize microtubules (Young et al., 2008; Bartolini and Gundersen, 2010; Chesarone et al., 2010; Földi et al., 2017). INF2 is a potent microtubule bundler *in vitro* and is capable of microtubule binding alongside F-actin, thus causing co-bundling of actin filaments and microtubules (Gaillard et al., 2011). Microtubule binding involves the FH1–FH2 domains of INF2, and binding of INF2 stabilizes microtubules. Actin filament and microtubule-bundling activities of inverted formins have been independently validated by genetic and *in vivo* analyses in *C. elegans*, which have demonstrated that the Form3 ortholog EXC-6 plays a key role in promoting interactions between F-actin and microtubules, and coordinates these two types of cytoskeleton during tubulogenesis in excretory cells (Shaye and Greenwald, 2015). Of note, recent findings also indicate that *Drosophila* Form3 is required for maintaining a stable microtubule population in dendritic cells via binding to microtubules through its FH1–FH2 domains. This function of Form3 appears to be crucial for dendritic arbor formation of these nociceptive sensory neurons and can be partially complemented by human INF2 (Das et al., 2021). Taking all these results together, we propose that the primary function of Form3 in tendon cells is to coordinate and stabilize the F-actin and microtubule arrays of their cytoskeletal belts. Form3 likely acts as a cytolinker that is essential for the parallel and rigid alignment of actin filament and microtubule bundles within the attachment plates. These crosslinking and stabilizing functions are crucial for the capacity of these cytoskeletal arrays to resist the muscle contraction forces. This proposed function aligns with our observation that, in *form3* mutants, the elongated F-actin fibrils and microtubule bundles are partially separated sideways (Fig. 6). Interestingly, some of these proposed functions of Form3, particularly as a microtubule stabilizer, are analogous to the proposed functions of the spectraplakin Shot in stabilizing the tendon cell cytoskeleton. Unlike Form3, Shot is also necessary for anchoring the cytoskeleton to the basal tendon cell junctions, and it is currently unclear whether Shot functions as an F-actin–microtubule crosslinker in this context (Lee and Kolodziej, 2002; Voelzmann et al., 2017). In addition, Shot localizes predominantly to the apical and basal ends of the cytoskeletal belt and appears to function mainly basally (Alves-Silva et al., 2008), whereas Form3 protein is enriched in the middle region (Fig. 7). Therefore, we propose that, different to Shot, Form3 performs its roles as a cytolinker and microtubule stabilizer by binding along the length of the actin filaments and microtubules within the mid-filament region of the cytoskeletal belt of the tendon cell attachment plate.

Related functions of Form3, potentially with cell type-specific modifications, are also likely to be relevant for stabilizing specialized F-actin and microtubule cytoskeletons during tracheal tube formation (Tanaka et al., 2004), in amnioserosa and dorsal epithelial cells during dorsal closure (Toth et al., 2022), in wing epithelia (Fig. S1) and in infiltrating astrocytes during axon pruning in the mushroom body of the CNS (Marmor-Kollet et al., 2023). As has been noted, another example of a cell type with rigid apicobasal F-actin and microtubule cytoskeletons, spectraplakins, myosin II, and 15-protofilament microtubules – all hallmarks of *Drosophila* tendon cells – are the support cells in the organ of Corti in the vertebrate inner ear (Saito and Hama, 1982; Slepecky and Chamberlain, 1983, 1985; Tucker et al., 2004; Alves-Silva et al., 2008). Therefore, it would be intriguing to determine whether INF2 is required for generating the rigidity of the cytoskeletal arrays of these support cells (and likewise, in vertebrate tendons) as well.

## MATERIALS AND METHODS

### Fly cultivation

*Drosophila* stocks were reared on standard fly food [see https://bdsc.indiana.edu/information/recipes/bloomfood.html but with 3 g Nipagin (Sigma-Aldrich, cat. no. H5501), 5 ml propionic acid and 12 ml 70% ethanol added per liter] at 25°C. Third-instar larvae were collected when crawling upwards out of the food. Both females and males were used for experiments.

### *Drosophila* stocks

*CD8-GFP-Shaker* (Zito et al., 1999); *form3^MI02995^* [BL-36174, *Bloomington* Drosophila Stock Center (BDSC)]; *form3^C265^* (this work); *form3^F958^* (this work; BL-92975, BDSC); *form3^F958^ sr^md710^* (this work); *form3^Em31^* (Tanaka et al., 2004; gift from Akinao Nose, The University of Tokyo, Japan); *form3^MI08774-GFSTF.2^* (*form3-GFP*; BL-65385, BDSC); P{y[+t*] w[+mC]=UAS-Lifeact-GFP}VIE-260B (BL-35544, BDSC); *Df(3L)BSC27* (BL-6867, BDSC); *Df(3L)Exel6110* (BL-7589, BDSC); *sr^md710^* (*stripe-Gal4*; BL-26663, BDSC); *P{w[+mC]=UASp-F-Tractin.tdTomato}15A* (BL-58989, homozygosed, BDSC); *P{w[+mC]=UASp-GFPS65C-alphaTub84B}14-6-II*; (BL-7374, homozygosed, BDSC); *P{w[+mC]=UAS-gapGFP}AC1* (BL-4522, BDSC; expresses GFP tagged with the myristylation sequence from GAP43); *TRiP.HMS00393attP2* (*form3* dsRNA; BL-32398, BDSC); *TRiP.HMS01978attP2* (*DAAM* dsRNA; BL-39058, BDSC); *TRiP.HMJ21037attP40* (*Fhos* dsRNA; BL-51391, BDSC); *TRiP.HMS00445attP2* (*Frl* dsRNA; BL-32447, BDSC); *TRiP.HMS00180attP2* (*mwh* dsRNA; BL-34862, BDSC); *P{w[+mC]=UASp-dia.EGFP}2* (GFP-tagged form; BL-56751, BDSC); *P{w[+mC]=UAS-dia.CA}3* (constitutively active form; BL-27616, BDSC).

### Recombinant DNA

pCFD5 (Port and Bullock, 2016; Addgene plasmid 73914); pCFD5_form3-gRNA (this work); pHD-2xHA-ScarlessDsRed (Kate O'Connor-Giles lab; *Drosophila Genomics Resource stock number 1366*); pScarless_form3-2xHA (this work).

### Mutation mapping of *F958* and *C265* through deficiency screen and exon sequencing

Alleles *F958* and *C265* were mapped by screening adults for the wing phenotype detected in transheterozygous point mutants, which showed held-out and bent-down wings *in trans* to third chromosome deficiencies. Three shorter deficiencies overlapping the region of the first hit (*Df(3L)BSC27*) were tested. *Df(3L)BSC224* (BL-9701, BDSC) and *Df(3L)Exel6110*, but not *Df(3L)374* (BL-24398, BDSC), showed a wing phenotype *in trans* to either of the two alleles. Alleles were next crossed to mutants available for three genes uncovered by the shortest deficiency generating the phenotype, *Df(3L)Exel6110* (Fig. 3A, black and red blocks). Mutants for *melted* (*melt^Δ1^*, gift from Aurelio Teleman, DKFZ, Heidelberg, Germany), *male- specific lethal 3* (*msl-3^1^*; BL-5872, BDSC) and *form3* (*form3^Em31^*; Tanaka et al., 2004; gift from Akinao Nose) were tested, and only *form3^Em31^* reproduced the wing phenotype *in trans* to both of our alleles. Both alleles, *F958* and *C265*, have wild-type wings *in trans* to deficiencies uncovering other formin members on the third chromosome, which are *mwh* (*Df(3L)ED4196*; BL-8050), *Frl* (*Df(3L)ED4543*; BL-8073), and *Fhos* (*Df(3L)BSC612*; BL-25687). Subsequently, overlapping PCR-amplified DNA fragments from *form3^F958^/w^1118^* and *form3^C265^/w^1118^* adults covering all *form3* exons were created (Table S5 for primer sequences). The individual fragments were each sequenced in both directions. Double base reads that were consistent between *F958* and *C265* were counted as single nucleotide polymorphisms present on the isogenized chromosome used for mutagenization. Double base reads that were unique to one of the two alleles were checked for consistency in the forward and reverse sequencing reads, which identified one non-silent point mutation each, unique to one of the two alleles.

### Protein domain border assignments for Formin 3

To locate the border of the FH2 domain we used the NCBI conserved domain database (CDD; Lu et al., 2020), SMART (Letunic et al., 2021) and Phyre2 (Kelley et al., 2015). Predicted FH2 domain secondary structures were compared to an alignment with homologous sequences from known and previously analyzed templates. Since the predictions did not match each other, we were only able to tentatively define the limits of the FH2 domain. The most N-terminal and most C-terminal amino acid that was predicted as part of the FH2 domain was used and annotated as the borders of the domain in this work (Table S3). The secondary structure predicted by Phyre2 is characterized by α-helices and conserved isoleucine and leucine residues (reported previously as vital for FH2 domain function in mammalian inverted formins; Hegsted et al., 2017). In the Phyre2 alignments, we also observed a significant number of conserved glutamic acid residues within the Form3 FH2 domain. The FH1 domain was manually annotated by searching for an accumulation of proline residues in the current Form3 protein sequence (*D. mel.* Release 6; https://flybase.org/; FBpp0099826 and FBpp0112380). Two or more prolines were counted as an accumulation. The first and the last proline residue in this structure was defined as one of the domain borders (Table S3) (Higgs and Peterson, 2005).

### Generation of HA-tagged *form3* allele

At the time of our experimental work, neither an antibody nor a functional tagged fusion protein was available for Form3. Hence, we introduced a 2×HA-tag at the endogenous C-terminal end of Form3 (Fig. 3; Fig. S10) via CRISPR-induced double-strand break and subsequent homology-directed repair to create *form3^HA^* (available as BL-92974, BDSC), using the strategy described at https://flycrispr.org/scarless-gene-editing. All related primers are listed in Table S5. Sequences for guide RNAs (gRNAs) were assembled on pCFD5 as described previously (Port and Bullock, 2016). *form3* homology sequences (3L:7121619.7122951 and 3L:7123023.7124407) were cloned into pHD-2xHA-ScarlessDsRed. Primers containing the gRNA guide sequences with pCFD5 (Addgene plasmid 73914) as template or primers for the generation of homology arms with genomic DNA as template were used with the Q5 Hot Start High-Fidelity 2× Master Mix (New England BioLabs, cat. no. M0494) to amplify desired PCR fragments. Fragments were gel-purified with the QIAquick Gel Extraction Kit (Qiagen, cat. no. 28704). Final vectors were assembled using the NEBuilder HiFi DNA Assembly Cloning Kit (New England BioLabs, cat. no. E5520) following the provided protocol. For pCFD5_form3-gRNA, 0.034 pmol purified gRNA containing PCR fragments was combined with 0.025 pmol BbsI-HF (New England BioLabs, cat. no. 3539)-digested pCFD5 backbone. For pScarless_form3-2xHA, 2 µg pHD-2xHA-ScarlessDsRed (*Drosophila Genomics Resource stock number 1366*) was digested with 10 U Bpu10I and 20 U PstI-HF (New England BioLabs, cat. no. R0649 and R3140) in 5 µl NEBuffer^TM^ 3.1 and a total reaction volume of 50 µl for 3 h at 37°C. Two fragments were cut out and purified from a gel with the QIAquick Gel Extraction Kit and subsequently combined with the two PCR amplified homology arms at an approximate ratio of 1:1:1:1 and a total of 0.37 pmol DNA. Potential bacterial transformants were analyzed by colony PCR using the Quick-Load Taq 2× Master Mix (New England BioLabs, cat. no. M0271). After sequencing, both plasmids were injected into embryos of flies expressing Cas9 in the germline (*y[1] M{GFP[E.3xP3]=vas-Cas9.RFP-}ZH-2A w[1118]*; BL-55821) (BestGene Inc. Chino Hills, CA, USA). Potential recombinants were selected by DsRed expression. DsRed was removed by crossing in pBac transposase (*Herm{3xP3-ECFP,alphatub-piggyBacK10}M6; MKRS/TM6B,Tb*; from BL-32070, BDSC). The removal was not completely scarless due to an error in the cloning design of the 3′ homology arm. As a result, about 150 nucleotides of pHD-2xHA-ScarlessDsRed vector residues remain, adding 20 spurious codons following the HA sequences before running into a stop codon (Fig. S10).

### Embryo collection, larval filet preparations and adult thorax sections

Embryos for antibody staining and *in situ* hybridization were formaldehyde-fixed according to standard protocols (Rothwell and Sullivan, 2007) and were not stored for longer than one week prior to *in situ* staining.

Larvae were collected in a mesh placed in cold PBS and immobilized on ice. One larva at a time was transferred to a drop of cold PBS containing 5 mM EGTA on a dissection dish, dissected essentially as demonstrated previously (https://doi.org/10.6084/m9.figshare.1344809.v2) and subsequently fixed for 30 min in 3.7% formaldehyde in PBS containing 5 mM EGTA. After washing in PBT (PBS, 0.1% Tween 20), filets were stained with antibodies.

For adult thorax sections, flies were collected in an empty glass vial and immobilized on ice or frozen at −20°C. Heads, abdomen and legs were removed in a cold dissection dish. About ten prepared thoraces with wings were collected on a cold drop of PBS containing 5 mM EGTA at a time before transferring them into fixative for 15 min at room temperature. After rinsing at least twice in PBT, thoraces were either stored for up to 48 h at 4°C in PBT or used right away to make transverse or sagittal cuts of the flight musculature. For cuts, thoraces were processed one at a time. After drying excess liquid from the thorax it was placed in a small drop of 100% glycerol on a glass slide. Then, the thorax was oriented with the ventral side up with the help of the still-attached wings to stabilize the thorax, and the glass slide was dipped into liquid nitrogen. The frozen thorax was cut after slight defrosting using a razor blade (∼5–15 s after taking it out of the nitrogen, such that the thorax neither shatters nor indents during cutting). For transverse sections, a straight ventral–dorsal cut was made approximately in the middle between the first and second leg pair. Sagittal sections were made parallel to the ventral midline. One half of the cut thorax was subsequently transferred to a glass vial with PBT. The collected thorax halves were either stained with phalloidin only or with antibodies and phalloidin (see below).

### Fluorescent antibody staining and live imaging

Embryos and larval or adult dissections were incubated in 10% bovine serum albumin (BSA) in PBT for 1 h and then incubated in the following primary antibodies for 1–3 nights at 4°C: rabbit anti-GFP (1:150; Rockland, cat. no. 600-401-215), rabbit anti-RFP (1:250; Rockland, cat. no. 600-401-379S), mouse anti-HA (12CA5, 1:600; gift from Alexandra Schambony, Friedrich-Alexander-Universität Erlangen-Nürnberg, Germany; visualized with biotinylated secondary antibodies as described for *in situ* hybridization), mouse anti-βPS integrin [1:300; deposited to the Developmental Studies Hybridoma Bank (DSHB), University of Iowa by D. Brower; DSHB Hybridoma Product CF.6G11], rabbit anti-β1-Tubulin (1:800; gift from Detlev Buttgereit, Marburg University, Germany), guinea pig anti-Shot (1:600; gift from Talila Volk, Weizmann Institute, Israel), guinea pig anti-Zipper (1:800; gift from Règis Giet, Université de Rennes, France). The following secondary antibodies were used generally in a 1:200 dilution: Alexa Fluor 488-conjugated goat anti-rabbit (Jackson ImmunoResearch, cat. no. 111-545-003), Cy3-conjugated goat anti-guinea pig (Jackson ImmunoResearch, cat. no. 106-165-003), Cy3-conjugated goat anti-mouse (Jackson ImmunoResearch, cat. no. 115-165-003), Alexa Fluor 555-conjugated donkey anti-rabbit (Abcam cat. no. Ab150074), biotinylated goat anti-mouse (Jackson ImmunoResearch, cat. no. 115-065-003). For F-actin staining, diluted phalloidin was added to the secondary antibodies. Phalloidin was conjugated either with Atto 488 (Sigma-Aldrich, cat. no. 49409), Atto 550 (Sigma-Aldrich, cat. no. 19083) or Atto 647N (Sigma-Aldrich, cat. no. 65906) and used at 1:1000 dilution. Stained specimens were embedded in Vectashield Mounting Medium (Vector Laboratories, cat. no. H1000), optionally with DAPI (cat. no. H1200).

Live-imaging through the cuticle of larvae carrying *CD8-GFP-Shaker*, expressing a fusion protein in all body wall muscles that localizes to the postsynaptic membrane of type I neuromuscular junctions and more weakly to muscle membrane, was performed as described previously (Zito et al., 1999). In brief, larvae were immobilized by heat shock in a 65°C water bath and mounted in a drop of 80% glycerol on a slide with the lateral side facing upwards.

### *In situ* hybridization

The DNA template to create a ∼450 bp probe for *form3* mRNA detection was synthesized using Q5 Hot Start High-Fidelity 2× Master Mix (New England BioLabs, cat. no: M0494) and primers listed in Table S5. The PCR product was purified with QIAquick PCR Purification Kit (Qiagen, cat. no. 28106). *In vitro* transcription was prepared on ice in a 20 µl reaction as follows: 2 µl 10× transcription buffer, 2 µl DIG RNA Labeling Mix (Roche, cat. no. 11277073910), 2 µl RNase Inhibitor (New England BioLabs, cat. no. M0307), 200 ng purified PCR product, 2 µl T7 RNA Polymerase (Roche, cat. no. 10881775001). After incubation at 37°C for 3–4 h, RNA was purified with RNeasy Mini Kit (Qiagen, cat. no. 74104). The probe was dissolved in hybridization solution (50% formamide, 5× SSC, 100 µg/ml salmon sperm DNA, 50 µg/ml heparin, 100 µg/ml *E. coli* tRNA, 0.1% Tween 20). For *in situ* staining, embryos were rehydrated and post-fixed in 10% formaldehyde in PBT for 20 min. After washing, embryos were incubated in hybridization solution for 1 h at 55°C. 1:500 diluted *form3* probe in hybridization solution was dispensed to the embryos and incubated for 2 days at 55°C. After two washes for 30 min each in hybridization solution at 55°C, twice for 30 min in 50% hybridization solution/PBT at 55°C, one rinse in PBT and two washes for 5 min in PBT at room temperature, embryos were incubated in sheep anti-digoxygenin (1:2000, Roche, cat. no. 11333089001) overnight at 4°C. After washing, embryos were incubated in biotinylated anti-sheep secondary antibodies (1:200, Vector Laboratories) for at least 2 h at room temperature. After washing, embryos were incubated in preincubated AB complex (1:100 A and 1:100 B from Vectastain Elite ABC Kit, Vector Laboratories, cat. no. PK-6100) for 1 h. For detection, washed embryos were incubated in TSA Fluorescein (1:100 in amplification diluent; Perkin Elmer, cat. no. SAT701001EA) and stained for 20 min in the dark. Stained embryos were embedded in Vectashield Mounting Medium (Vector Laboratories, cat. no. H1000).

### Microscopy

Confocal *Z*-stacks of live-imaged or fixed specimens were acquired with a Leica SP5 II (10× Air, 20×/0.7 HC PL APO Glycerol or 63×/1.3 HC PL APO Glycerol objectives). Additional images were acquired with a Zeiss Axio Imager.Z1 equipped with an ApoTome and 20×/0.8 Plan-Apochromat; 40 × 1.3 Plan-Apochromat Oil objectives. Projections of the *Z*-stacks and re-slices were processed with FIJI/ImageJ (v2.9.0/1.53t) (Schindelin et al., 2012). Stacks of three to five muscle tips were scanned and evaluated for each marker combination of mutants shown in Figs 6 and 7 at 63× magnification and with sequential scans for the different channels. An additional ∼30–120 muscles/tendons were scanned and examined at 20× or 10× objective magnification. Embryo stainings and larval live specimens carrying Form3–GFP were scanned with a Zeiss LSM710 confocal system, using 20× air objectives (10× for third-instar larvae). For live imaging, larvae were raised on apple juice agar at 25°C with yeast, collected at the appropriate time point, heat shocked and mounted in 70% glycerol.

### Quantification and statistical analysis

Fully captured abdominal hemisegments from *form3^F958^/Df(3L)BSC27* larvae and controls heterozygous for *form3^F958^* over a complementing allele from the same EMS screen were analyzed for the phenotype of lateral somatic muscles. Muscles 5, 12, 21, 22 and 18 were analyzed individually. Muscles 23 and 24 were treated as a pair since these muscles could not readily be distinguished in affected hemisegments. The abundance of muscles that were shortened, malformed, had spiky tips or were located at abnormal positions (‘abnormal’) versus normally formed and positioned muscle fibers (‘normal’) was counted, and for a given muscle (or pair), the mean frequency of phenotypic muscles was calculated (Table S2). Fisher's exact test was used to determine whether the distribution of phenotypic versus normal muscles was dependent on the genetic background. Dependency was accepted at *P*≤0.05 and defined as highly significant at *P*≤0.001 (Fig. 2F; Table S2).

## Supplementary Material



10.1242/joces.263543_sup1 Supplementary information
