# Supplementary material for: Formin 3 stabilizes the cytoskeleton of Drosophila tendon cells, thus enabling them to resist muscle tensile forces
Source: J Cell Sci. 2025 Apr 15;138(7):jcs263543. doi: 10.1242/jcs.263543 (PMC12045603; doi:10.1242/jcs.263543)
Supplement: Supplementary information [file joces-138-263543-s1.pdf]

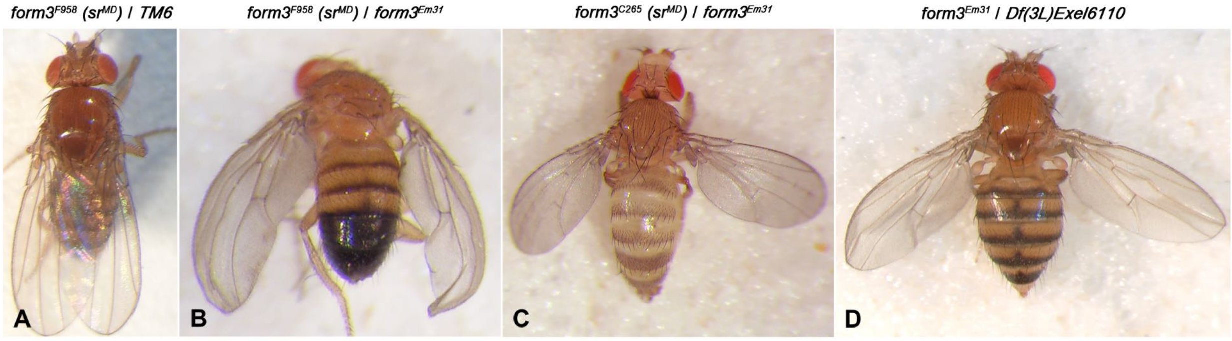

Fig. S1. Wing phenotypes of form3 mutants.

(**A**) Heterozygous control fly, *form3*^{F958} *sr*^{MD710}/*TM6* (*sr*^{MD710} bracketed as it is not functionally relevant in this context). (**B**) *form3*^{F958} *sr*^{MD710}/*form3*^{Em31} transheterozygous fly showing held-out wings that are bend downwards and wrinkled. (**C**) *form3*^{C265} *sr*^{MD710}/*form3*^{Em31} transheterozygous fly showing held-out wings that are bend downwards. (**D**) *form3*^{Em31}/*Df(3L)Exel6110* transheterozygous fly showing held-out and wrinkled wings.

Journal of Cell Science • Supplementary information

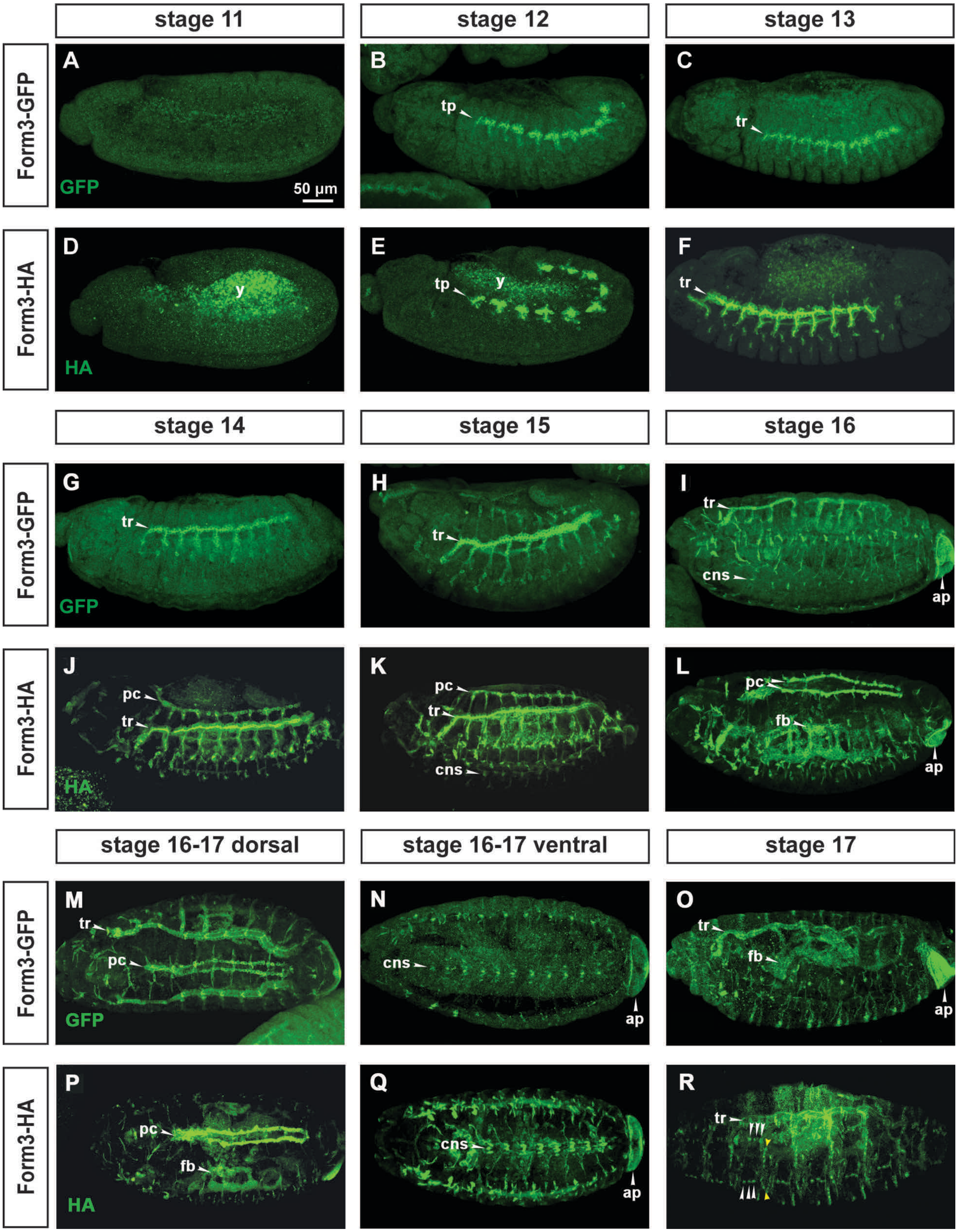

Fig. S2. Embryonic expression of Form3: Comparison of spatial and temporal expression patterns of Form3-GFP and Form3-HA.

Fixed embryos were stained with GFP and HA antibodies. Embryos are oriented such that anterior is to the left and (in side views) dorsal is up. (**A, D**) Until stage 11 there is no spatially

Journal of Cell Science • Supplementary information

distinct expression of either of the two tagged Form3 versions (signals in yolk are unspecific). (**B**, **E** to **O**, **R**) From stage 12, both tagged Form3 versions are expressed in tracheal precursors and tracheal cells. (**K**, **N**, **Q**) Expression in specific medial neurons of the central nervous system occurs from stage 15 until the end of embryogenesis. Expression in pericardial cells starts from stage 14-15 (**J**, **H**, **K**, **L**, **M**, **P**,) and in the fatbody from stage 15 (**K**, **I**, **L**, **M**, **P**, **Q**, **O**, **R**). (**P**) shows the only embryo (st. 17) found with expression in tendon cells (white arrowheads: tendon cells of lateral transverse muscles; yellow arrowheads: tendon cells at segment borders). Abbreviations: cns: central nervous system; fb: fatbody; pc: pericardial cells; tp: tracheal precursors; tr: trachea; y: yolk. Scale bar: 50µm.

Journal of Cell Science • Supplementary information

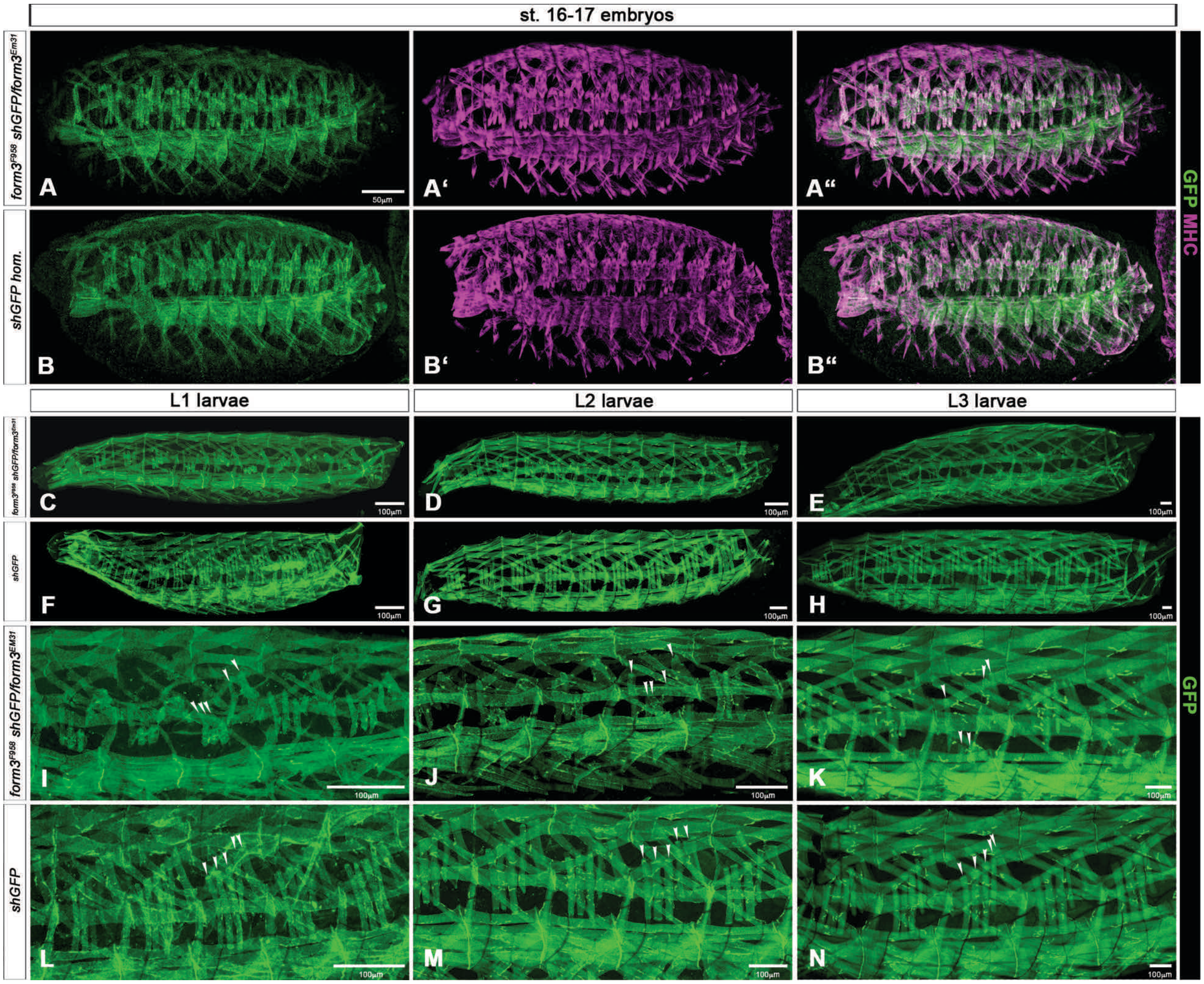

Fig. S3. Analysis of *form3* mutant muscle phenotypes in late stage embryos and during different larval stages.

Phenotypes were analyzed in *form3^{F958} shGFP (= CD8-GFP-Shaker)/form3^{Em31}* animals and compared to homozygous *shGFP* animals, as indicated on the left. Color codes of the labels are shown on the right. Fixed embryos (**A** - **B"**) were stained with GFP (green) and MHC (myosin heavy chain, magenta) antibodies whereas larvae were live imaged for GFP signals (**C** - **N**). (**A** - **A"**) The muscle pattern of a stage 16-17 embryo, visualized via GFP (**A**, **A"**) and MHC (**A'**, **A"**), is identical to the control (**B** - **B"**). (**C**, **D**, **E**, **I**, **J**, **K**) The lateral transverse muscles (LTMs) and muscle 18 (M18) (arrowheads) of 1st instar (L1) larvae, 2nd instar larvae (L2) and 3rd instar larvae (L3) are shortened and mis-shapen as compared to the controls (**F**, **G**, **H**, **L**, **M**, **N**).

Scale bars: (A - B"): 50µm: (C - N): 100µm.

Journal of Cell Science • Supplementary information

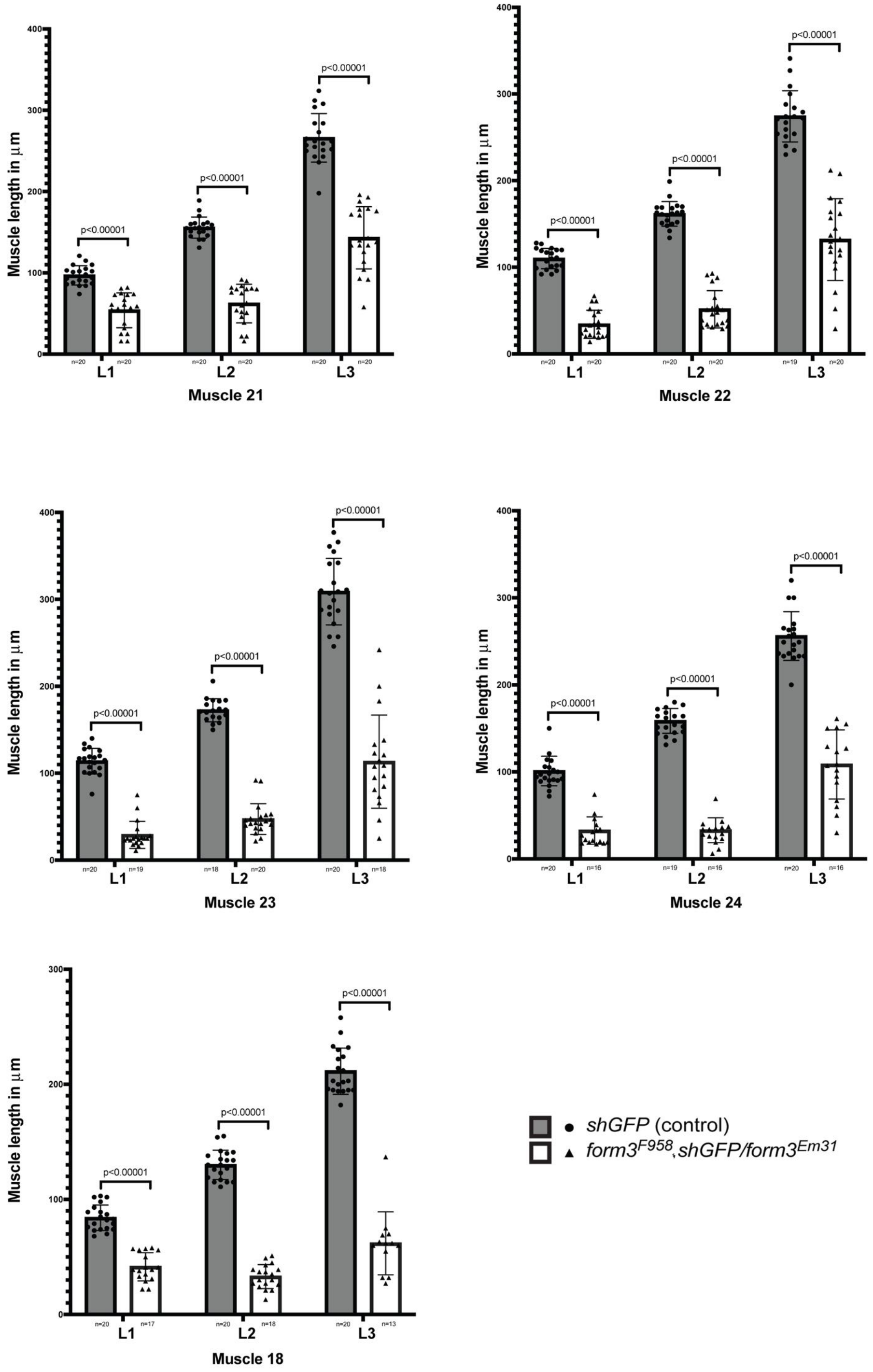

Fig. S4. Lengths of lateral transverse muscles 21 to 24 and of muscle 18 in the three larval stages (L1: 1st instar, L2: 2nd instar; L3, 3rd instar).

Muscle lengths of *form3*^{F958} *shGFP (= CD8-GFP-Shaker)/form3*^{Em31} mutant larvae and homozygous *shGFP* control larvae were measured with Fiji using the "Straight line" tool. 20 hemisegments from four larvae per genotype (normally from abdominal segments A2-A6) were measured. Muscles that could not be clearly identified were excluded. After checking the normality of the data, two-tailed t-tests or Mann-Whitney tests were used to determine p-values.

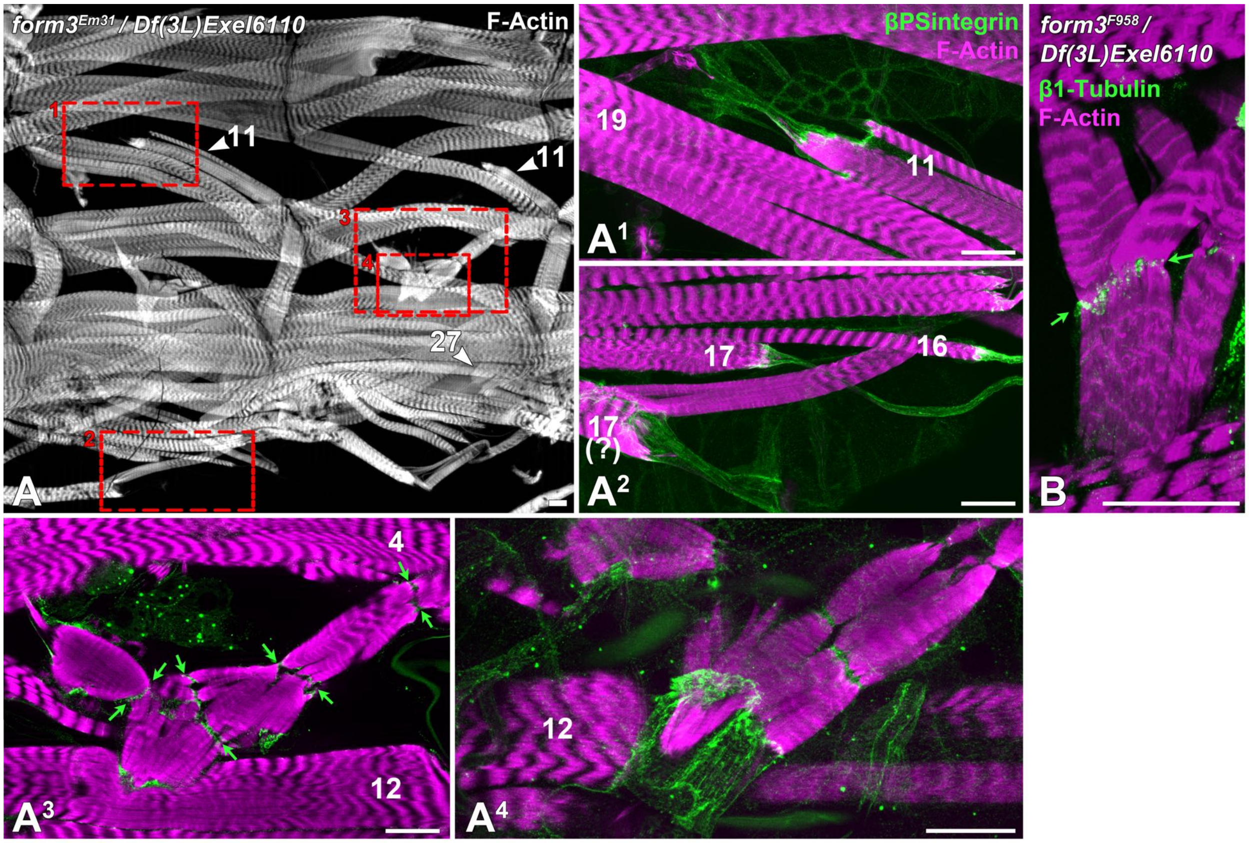

Fig. S5. Loss of *form3* results in failure of tendon cell integrity and muscle retraction at direct myotendinous junctions.

3rd instar larvae transheterozygous for *form3*^{Em31} (**A** - **A**^{4}) or *form3*^{F958} (**B**) and *Df(3L)Exel6110* shows abnormalities in several muscles that coincide with abnormally shaped tendon cells at their attachment sites. Muscles are stained for F-actin (grey, magenta) and in (A^1 - A^4) tendon cells plus muscle tips are visualized with antibodies against βPSintegrin (green). In (B) tendon cells or fragments thereof are labeled with β1-Tubulin antibodies (green). Muscles 4, 11, 12, 19, and 27 are marked. Anterior is left, dorsal is up, view from exterior. (**A**) Somatic muscles 11, lateral muscles 21-24 and 18, and ventral muscles 15-17, 27 and 29 show abnormally pointy tips, are shortened, wrongly oriented, and/or attached at abnormal positions or ectopically to each other in a *form3* loss-of-function background. (**A**^{1}) The βPSintegrin-positive tendon cell at the retracted anterior tip of M11 is elongated correspondingly, resulting in a tube-like instead of the typical crescent shape. (cf. Fig. 3). (**A**^{2}) Tendon cells at the posterior attachment sites of ventral oblique muscles also show severely elongated phenotypes. (**A**^{3}) At abnormal muscle-to-muscle attachments between mis-positioned lateral transverse muscles, βPSintegrin accumulates in thin lines (green arrows), presumably corresponding to ectopic muscle-to-muscle attachments with possible contribution of tendon cells remnants. (**A**^{4}) Higher Z-planes and enlarged as compared

Journal of Cell Science • Supplementary information

to (A^3), showing that at the ventral end of the chain of lateral muscles βPSintegrin marks a stretched cell corresponding to an abnormally elongated tendon cell. Scale bars: 25 μm. (**B**) β1-Tubulin accumulates at a presumed ectopic muscle-to-muscle attachment (green arrows) between shortened, abnormal lateral muscles, probably corresponding to remnants of ruptured tendon cells. Images in (A – A4) were scanned with a Zeiss Apotome and image in (B) with a Leica SP5 II confocal system.

Journal of Cell Science • Supplementary information

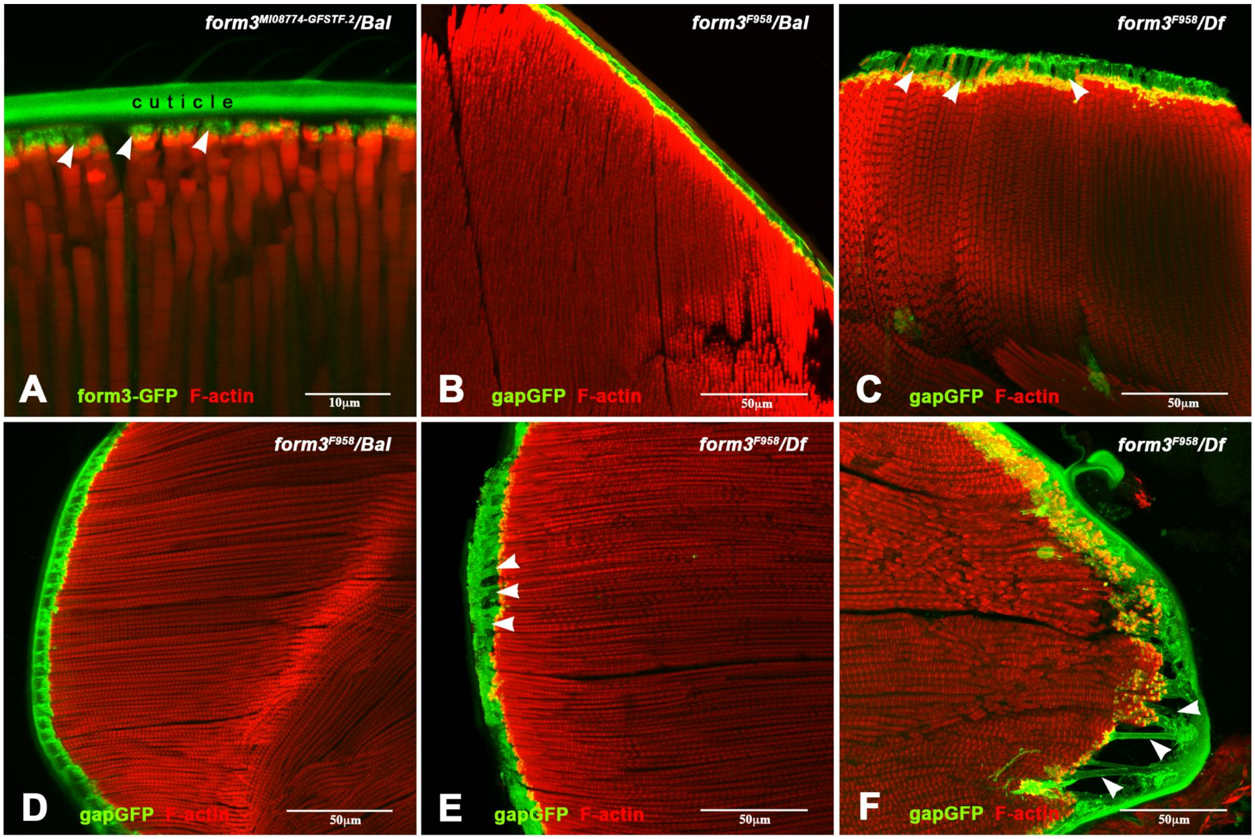

Fig. S6. Expression and function of Form3 in thoracic tendon cells of indirect flight muscles.

(**A**) In a fly carrying the gene trap insertion *form3*$^{MI08774\text{-}GFSTF.2}$ *in trans* to a *TM3* balancer chromosome, expression of the Form3-GFP fusion protein (green) is seen in the tendon cells of the dorsoventral indirect flight muscles (red) (cuticle shows autofluorescence; scale bar: 10µm). (**B** – **F**) Morphologies of tendon cells and indirect flight muscles in controls (B, D; genotype *UAS-gapGFP*/+; *form3*^{F958} *srMD*/*TM3*) and *form3* mutant escaper flies (*UAS-gapGFP*/+; *form3*^{F958} *sr*^{MD}/*Df(3L)Exel6110*) (gapGFP is a GFP version tagged with the myristylation sequence from GAP43, see M&M). For simplicity, only the genotypes relevant to *form3* function

Journal of Cell Science • Supplementary information

are shown in labels. Scale bars: 50µm. (**B**) Dorsal tendon cells labeled with gapGFP in control fly are arranged as a regular, thin layer of cuboidal cells to which the dorsoventral indirect flight muscles are attached. (**C**) Dorsal tendon cells in a *form3* mutant escaper fly are variably stretched, with the attached flight muscles being retracted correspondingly, as compared to the control. (**D**) Anterior tendon cells labeled with gapGFP in control fly are arranged as a regular layer of cuboidal cells to which the dorsal longitudinal indirect flight muscles are attached. In *form3* mutant escaper flies (**E**, anterior thorax; **F**, posterior thorax) the tendon cells are variably stretched, with the attached flight muscles being retracted correspondingly, as compared to the control. In all panels GFP was detected via GFP fluorescence and F-actin was stained with phalloidin.

Journal of Cell Science • Supplementary information

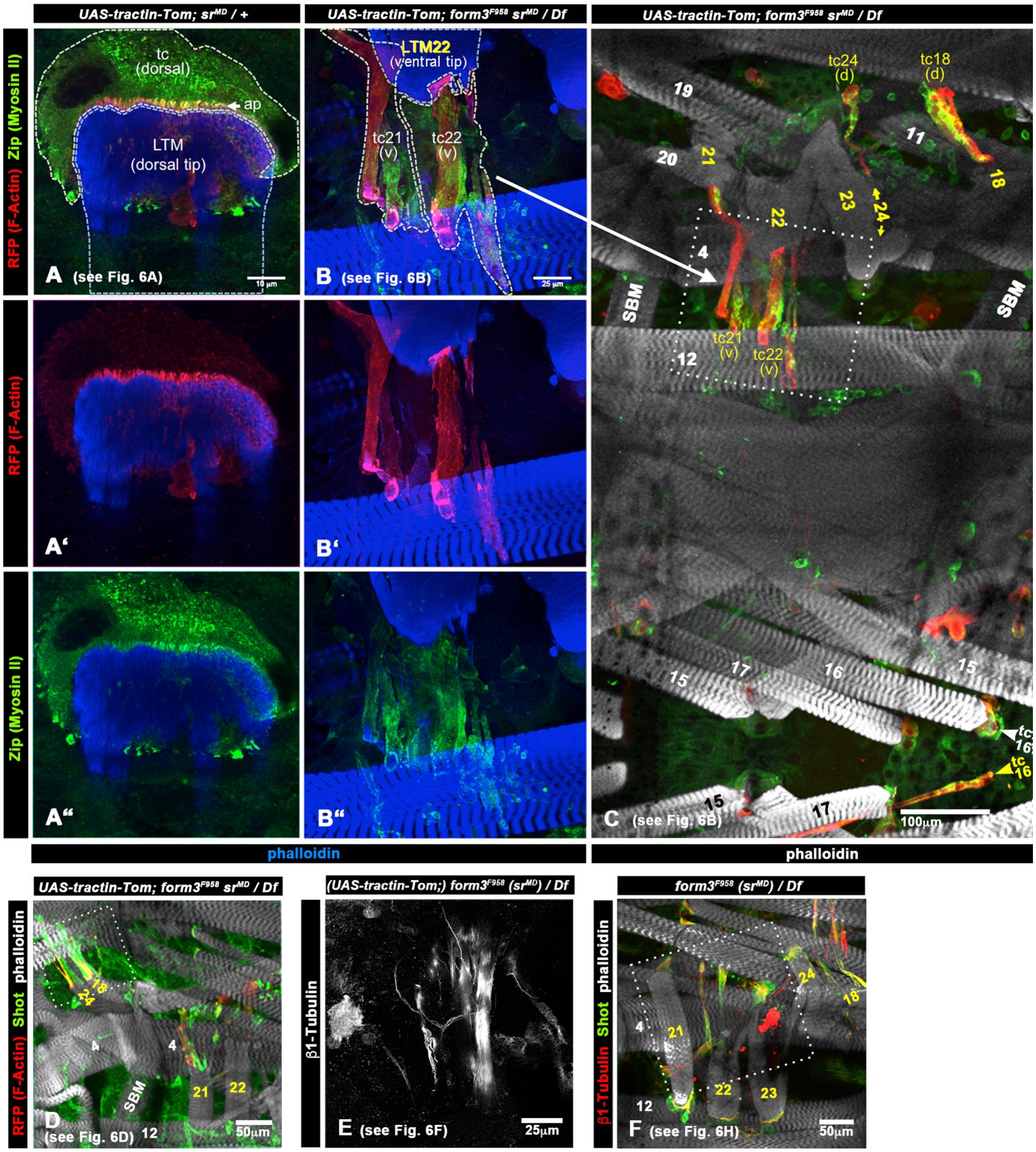

Fig. S7. Supplement to Fig. 6, showing cell outlines, single channels, and surrounding muscle contexts.

In all images, dorsal is up and ventral is down. Genotypes are shown on the top and the color codes for the probes on the left and bottom of the panels. Affected muscle and tendon cells (tc) are labeled in yellow. (**A** - **B”**) Shown are the outlines (dotted) of the lateral transverse muscles (LTM) and tendon cells (tc) depicted in Fig. 6A (control) and Fig. 6B (*form3* mutant), respectively. In (A - A”) the dorsal tip of an LTM (scale bar: 10 µm) and in (B - B”) the ventral tip of an LTM22 (scale bar: 25µm) is shown. (**C**) The dotted square shows the location of the structures depicted in (A - B”) within the muscle context of the entire segment (between segment

border muscles, SBM). Scale bar: 100μm. (**D**) The dotted square shows the location of the structures within surrounding muscles depicted in Fig. 6D. Scale bar: 50 μm. (**E**) Shown is the single channel image for β1-Tubulin, as depicted in Fig. 6F in red. Scale bar: 25 μm. (**F**) The dotted square shows the location of the structures within surrounding muscles depicted in Fig. 6F. Scale bar: 50 μm.

Journal of Cell Science • Supplementary information

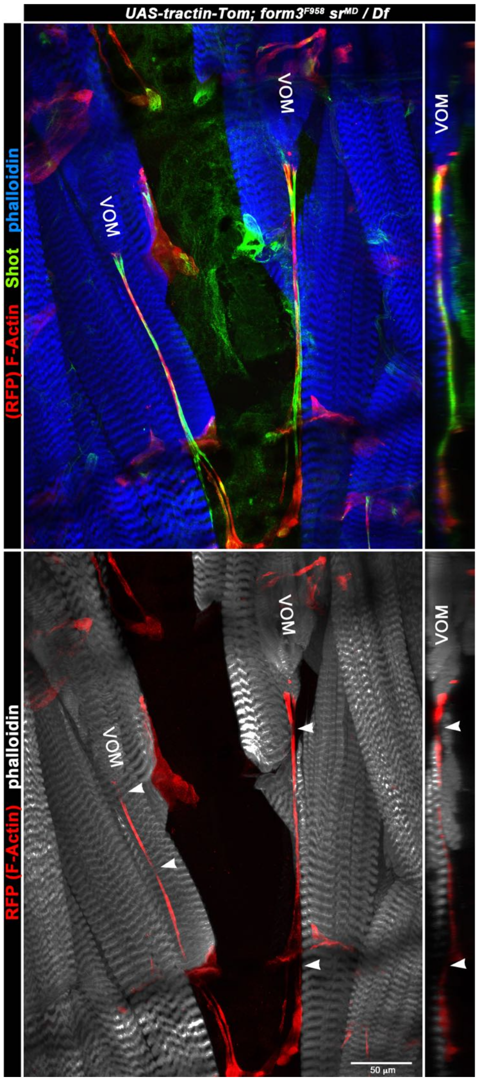

Fig. S8. Morphological and cytoskeletal defects of tendon cells of directly attached ventral oblique muscles in *form3* mutant.

The tendon cells of a ventral oblique muscle (VOM, likely M16 or M17), in a 3rd instar larva of the genotype *UAS-tractin-Tom*/+ ; *form3^{F958} srMD*/*Df(3L)Exel6110* is extremely elongated, with the attached VOM being balled up concomitantly. F-actin fibrils (red, stained for tractin-Tomato with anti-RFP) extend throughout the length of the extended tendon cells but show interruptions at several positions (arrow heads in bottom panels). Shot (green, stained with anti-Shot) is enriched at these positions. (left hand panels, XY projections in Z; right hand panels, YZ projections in X of muscle on right side of larva; F-actin in muscles stained with phalloidin; scale bar: 50 µm).

Journal of Cell Science • Supplementary information

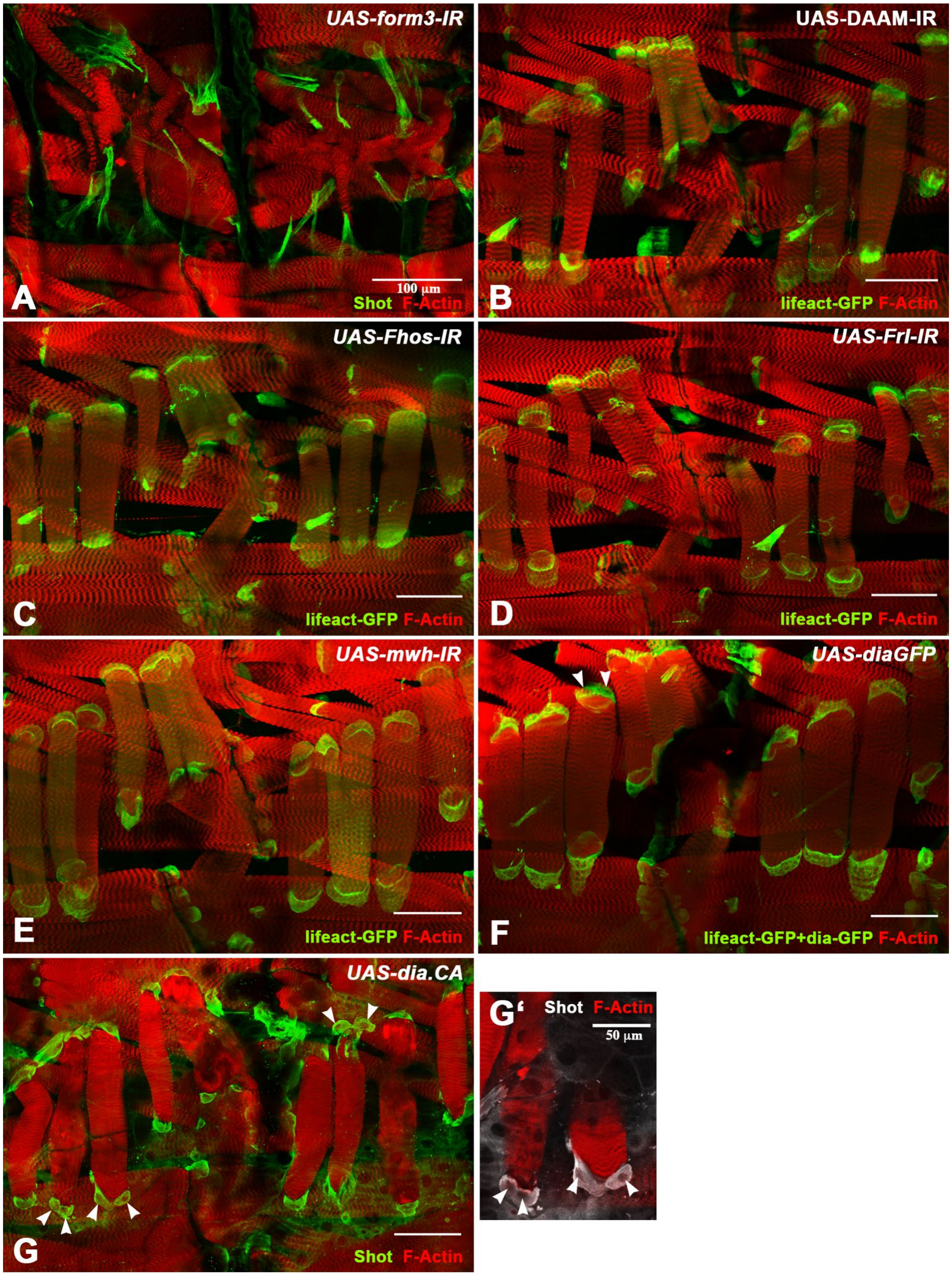

Fig. S9. Examination of the roles of different formin family members in larval tendon cells. Shown are the lateral muscles of 3rd instar larvae expressing the respective agents under UAS control by *sr-GAL4* (*sr*^{MD710}) and stained for tendon cells (green) as well as muscles (phalloidin, red). In (B) to (F) *UAS-Lifeact-GFP}VIE-260B* was present additionally on one of the 2nd chromosomes. (**A**) RNAi knock-down of *form3* gives a strong tendon cell phenotype, as detected with anti-Shot and phalloidin, similar to *form3* null mutants. Among 20 scanned LTMs and five

Journal of Cell Science • Supplementary information

M18, 100% were severely affected. (**B** – **E**) Upon knock-downs of the Formin genes *Dishevelled Associated Activator of Morphogenesis* (*DAAM*), *Formin homology 2 domain containing* (*Fhos*), *Formin-like* (*Frl*), and *multiple wing hairs* (*mwh*), no extensions of tendon cells (stained with anti-GFP) are observed. (**F**, **G**) Forced tendon cell expression of functional Diaphanous (Dia)-GFP fusion protein (**F**) and a constitutively active version of Dia, Dia.CA (**G**) (see Fig. 3A for control) do not induce stretched tendon cells and contracted muscles. However as indicated by their nuclei (GFP-negative) many attachments consist of a pair instead of a single tendon cell (arrow heads), suggesting that overactivity of Dia can induce an additional round of cell division during tendon cell development (see also (Sansores-Garcia et al., 2011)). (G') Higher magnification view of the LTMs from the left side of (G) with Shot shown in white and the arrowheads pointing to the nuclei (spared of Shot) of the two tendon cells at each attachment. Scale bars: (A - G) 100 μm, (G') 50 μm.

Journal of Cell Science • Supplementary information

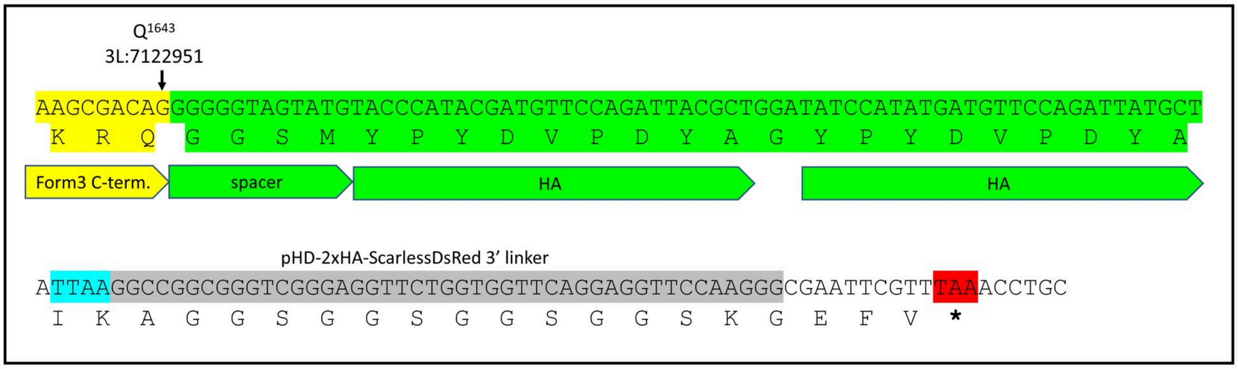

Fig. S10. 3' end of *form3*^{HA} with predicted translation.

Q1643 (isoform A) is the second of the last amino acid of Form3 and is fused to the 2xHA tag. dsRed was recombined out, leaving a single TTAA (turquoise). This site continues with ~150 ntd. from the 3' end of the pHD-2xHA-ScarlessDsRed vector, starting with its 3' linker sequence, which adds spurious codons to the HA tag sequences before a stop codon (red).

Journal of Cell Science • Supplementary information

Table S1. Ratio of transheterozygous adults of alleles *F958* and *C265 in trans* to each other and to three different deficiencies. Flies with the respective mutations shown in the table, each balanced over *TM6*, were crossed to each other. Shown are the numbers and percentages of eclosed transheterozygotes compared to the total number of eclosed flies. Full complementation would be expected to yield 33 % transheterozygotes.

	C265	***Df(3L)BSC27***	***Df(3L)BSC224***	***Df(3L)Exel6110***
F958	52/304 (17 %)<br>bent-down wing phenotype	7/72 (10 %)<br>strong wing phenotype	7/37 (19 %)<br>wing phenotype	12/147 (8 %)<br>strong wing phenotype
C265	n.d.	7/23 (30 %)<br>mild wing phenotype	29/110 (26 %)<br>wings bent and not folded on edges, milder phenotype	53/252 (21 %)<br>mild wing phenotype
EM31	n.d.	n.d.	n.d.	55/344 (16 %)<br>Strong wing phenotype

Table S2. Frequency of muscle phenotype in *form3*^{F958}*/Df(3L)BSC27* larvae and controls.

	Muscle 18	Muscle 21	Muscle 22	Muscles 23/24	Muscle 5	Muscle 12
form3^{F958}*/ Df(3L)BSC27* (N=11)	92.6 % (25/27)	85.2 % (23/27)	88.9 % (24/27)	100 % (27/27)	0 % (0/24)	0 % (0/22)
form3^{F958}*/x** (N=22)	0 % (0/82)	0 % (0/82)	3.7 % (3/82)	3.7 % (3/82)	0 % (0/93)	0 % (0/46)
p from Fisher's exact test	1.25×10^{-22}	7.61×10^{-20}	9.33×10^{-18}	1.46×10^{-22}	1	1

Journal of Cell Science • Supplementary information

Table S3. (Tentative) assignments of Formin3 protein domain borders. Form3 domains include a diaphanous inhibitory domain (DID), a dimerization domain (DD), and the formin homology domains 1 and 2 (FH1/2).

	Gene (CDS)	DID	DD	FH1	FH2
form3	3L:7,094,818-3L:7,123,297 (7,094,893-7,122,957)	7,094,893-7,118,014	7,118,057-7,118,402	7,118,526-7,118,660	7,118,718-7,120,421
Form3-A	1644 aa	M1-S158 (158 aa)	V173-D229 (57 aa)	P271-P315 (45 aa)	A335-R843 (509 aa)
Form3-B	1717 aa	M1-S231 (231 aa)	V246-D302 (57 aa)	P344-P388 (45 aa)	A408-A916 (509 aa)

Table S4. Overview of *formin3* mutation and insertion locations. Exact genomic locations of point mutations and insertions in the *form3* locus with affected amino acids and resulting protein lengths of two isoforms Form3-A and -B are given. *F958* and *Em31* mutations introduce a premature stop codon and thus lead to a truncated peptide. The *C265* allele converts glycine to glutamic acid within the tentative FH2 domain borders. A GFP-tag from the Minos collection *MI8774* is inserted between exons 6 and 7.

	F958	***EM31***	***C265***	***MI8774***
form3	7,117,988 (CAG>TAG)	7,118,997 (CGA>TGA)	7,120,291 (GGA>GAA)	7,119,326 (intron)
Form3-A	Q150 (149 aa)	R428 (427 aa)	G800 (1644 aa)	
Form3-B	Q223 (222 aa)	R501 (500 aa)	G873 (1717 aa)	

Table S5. Oligonucleotides.

Genomic primers were designed with NCBI Primer-BLAST (Ye et al., 2012). Primers for vector sequencing were designed Primer3-based primer design tool from Benchling Inc. All primers were synthesized by metabion international AG. Primer sequences are listed in the following tables.

Primers for *in situ* probe synthesis (T7 promotor sequence in red)

Form3_F1	GAAGGTGGGTCGCCACATAA
Form3_R1_T7	GGATCCTAATACGACTCACTATAGCTCCTCTTGACCGTTGCAGT

Primers for *F958* and *C265* mutation mapping

form3_ex1_F	CTCACTGTGTGCCTCTCTGTTA
form3_ex1_R	ATCTCAGCTGACCGCGACTAA
form3_ex2-4_F	CAGCTTGAACCCAACTAACTG
form3_ex2-4_R	TGACTTTTGCGGGCTTATCG
form3_ex5_F	TTTTGTAGTGTAAGTCAAAGATCCC
form3_ex5_R	CACGATCGCGTCGAACTATG
form3_exB5.1_F	TGCGCAAGGCTAACGATTTT
form3_exB5.1_R	CAACGACAAAAGGGGGTGGG
form3_ex6_F	AACTACAAATTGCCAGCAGCA
form3_ex6_R	GATTCTCTTGCATGTTCATAGCC
form3_ex7a_F	CTGGCATTGTGGTTGGCTATG
form3_ex7a_R	TTCAGACTTCGCTTGCCGTC-
form3_ex7b8_F	GCTCCCAAGGCCAAGATGAA
form3_ex7b8_R	GGATTCACTGACTTGCTGGCAC
form3_ex89_F	TACAAATTGCGGATCGAGAGC
form3_ex89_R	GTCTTGTTCCAGGCGGATGA

Journal of Cell Science • Supplementary information

form3_ex1011_F	TCGGAGCATTCCTTCTTGGG
form3_ex1011_R	GCCGGACTCCTGATCCATTC
form3_ex12a_F	TGTTCGCAGAAAACGAACGA
form3_ex12a_R	TCCTCATTGCCACTGCTGTA
form3_ex12b_F	TGCTCAGTCACCACAAGACC
form3_ex12b_R	AGGAGCGCAGACTGTTTCTC
form3_3UTR_F	ACTGCAACGGTCAAGAGGAG
form3_3UTR_R	ACACACTTTGTAAACAACGCACT

Primers for generation of pScarless_form3-2xHA (overhangs for Gibson assembly in green, primers used for colony PCR marked with asterisk, primers used for sequencing marked with circle)

5hom_form3_fwd*	GGTGGATCTGGAGGTTCCGGCGGCCACGGTGAGCATGACGGATA
5hom_form3_rev*	ATCGTATGGGTACATACTACCCCCCTGTCGCTTGGTGGCACT
3hom_form3_fwd*	AGGGCGAATTCGTTTAAACCTGCAAACGCGAATGCATCCAACTC
3hom_form3_rev*	CTCACTAAAGGGACTAGTCCTGCATGTCCACGTTGGTTACCACTC
Seq_3hom_F°	CCAAGCGGCGACTGAGATGTCC
Seq_5hom_int*°	TAAACGAGACTCCTGCCCGCCA
Seq_5hom_R°	CTTCAGCTTGGCGGTCTGGGTG
dsRed-fwd°	TGATGAACTTCGAGGACGGC
dsRed-rev*°	GCTCCCAGCCCATAGTCTTC
M13-fwd°	GTAAAACGACGGCCAGT
M13-rev°	CAGGAAACAGCTATGAC

Guide RNA (gRNA) guide sequences were selected with the flyCRISPR Target Finder (Gratz et al., 2014). Only results with zero off-targets and an adjacent genomic protospacer adjacent motif (PAM) in the form NGG were considered. Suggested guide sequences were screened for reported

Journal of Cell Science • Supplementary information

single nucleotide polymorphisms (SNPs) with the FlyVar database (FlyVar 2014). Only guides with zero reported SNPs at the genomic binding site were selected to exclude potential mismatches between gRNA guide sequence and the genomic target site in the fly stocks used for CRISPR transgenesis.

gRNA guide sequences

	Sequence	PAM	Genomic target site
gRNAf1	ACTGGGGTTCGCACTCATCC	TGG	3L:7122848..7122870 (- strand)
gRNAf2	GTAAACTTGGGACGTTAACT	TGG	3L:7122990..7123012 (- strand)

Gratz, S. J., Ukken, F. P., Rubinstein, C. D., Thiede, G., Donohue, L. K., Cummings, A. M. and O'Connor-Giles, K. M. (2014). Highly specific and efficient CRISPR/Cas9-catalyzed homology-directed repair in *Drosophila*. *Genetics* 196, 961-71.
Sansores-Garcia, L., Bossuyt, W., Wada, K., Yonemura, S., Tao, C., Sasaki, H. and Halder, G. (2011). Modulating F-actin organization induces organ growth by affecting the Hippo pathway. *EMBO J* **30**, 2325-35.
Ye, J., Coulouris, G., Zaretskaya, I., Cutcutache, I., Rozen, S. and Madden, T. L. (2012). Primer-BLAST: a tool to design target-specific primers for polymerase chain reaction. *BMC Bioinformatics* **13**, 134.

Journal of Cell Science • Supplementary information
