# Supplementary material for: Formin 3 stabilizes the cytoskeleton of Drosophila tendon cells, thus enabling them to resist muscle tensile forces
Source: J Cell Sci. 2025 Apr 15;138(7):jcs263543. doi: 10.1242/jcs.263543 (PMC12045603; doi:10.1242/jcs.263543)
Supplement: Supplementary file 1 [file joces-138-263543_review_history.pdf]

Formin 3 stabilizes the cytoskeleton of *Drosophila* tendon cells, thus enabling them to resist muscle tensile forces

Helena Pissarek, Na Huang, Leanna H. Frasch, Hermann Aberle and Manfred Frasch

DOI: 10.1242/jcs.263543

Editor: Richa Rikhy

Review timeline

Original submission:	26 September 2024
Editorial decision:	28 October 2024
First revision received:	4 March 2025
Accepted:	6 March 2025

Original submission

First decision letter

MS ID#: jcs.263543

MS TITLE: Formin 3 Confers Cytoskeleton Resilience Against Muscle Tensile Forces in Drosophila Tendon Cells

AUTHORS: Manfred Frasch; Helena Muster; Leanna H. Frasch; Hermann Aberle

ARTICLE TYPE: Research Article

Dear Dr Frasch,

We have now reached a decision on the above manuscript.

To see the reviewers' reports and a copy of this decision letter, please go to:

As you will see, the reviewers gave favourable reports but raised some critical points that will require amendments to your manuscript. I hope that you will be able to carry these out because I would like to be able to accept your paper, after the comments of the reviewers are addressed.

Please ensure that you clearly highlight all changes made in the revised manuscript. Please avoid using 'Tracked changes' in Word files as these are lost in PDF conversion.

I should be grateful if you would also provide a point-by-point response detailing how you have dealt with the points raised by the reviewers in the 'Response to Reviewers' box. Please attend to all of the reviewers' comments. If you do not agree with any of their criticisms or suggestions please explain clearly why this is so.

© 2025. Published by The Company of Biologists under the terms of the Creative Commons Attribution License (https://creativecommons.org/licenses/by/4.0/).

Reviewer 1

SUMMARY OF THE ADVANCE MADE IN THIS PAPER AND ITS POTENTIAL SIGNIFICANCE TO THE FIELD

The manuscript by Muster, et al., is interesting as the authors define a new role for Formin 3 (Form3) in maintaining muscle-tendon attachment in larval tendon cells. Alleles corresponding to form3 were uncovered in an EMS screen and mutants homozygous for form3 show elongated tendon cells and a variety of aberrant muscle morphologies in muscles that are directly attached to the larval cuticle. Analysis of Form3 protein expression shows an enrichment in bundled arrays that span the length of the tendon cell. The reported data is appropriate for publication in JCS after revisions are made to bolster experimental conclusions, tighten up/clarify the manuscript writing, and modify the title to better reflect the results.

SUGGESTIONS TO AUTHORS
Essential tasks
(1) The title must be modified as the paper does not show that force directly impacts Form3 function and/or expression in tendon cells.
(2) Two review papers are used as references in the first intro paragraph. Schweitzer, 2010 is quite old and it would be better for readers to refer the original manuscripts, especially for bidirectional crosstalk between muscles and tendons during embryonic development.
(3) As currently written, the references for the ZPD proteins imply a direct role in embryonic and/or larval muscle-tendon attachments. Wilkin, et al., contains no functional data for Dumpy in this process. Bokel, et al., looks at wing and embryo while Chu and Hayashi examines IFMs. Please modify accordingly as this reviewer is unaware of a direct role for ZPD proteins in larval MAS. Accordingly, can ZPDs be conclusively shown the Fig. 1?
(4) Please renumber the figures, tables, etc. so they are referred to in order in the manuscript.
(5) Add the lethality data (p. 13, lines 39-54) to Table S5. Also, on p. 14, lines 20-24, include a comparison of Em31 in the allelic strength discussion.
(6) The sequencing results and domain descriptions would be easier to read (p. 15-16) if the domains/protein characteristics were all discussed first followed by the lesions identified. What about a lack of domains in the C-terminus (maybe mention this). Is the lack of a DAD conserved in mammalian formin3 proteins? Is it worth showing comparisons to other organisms due to these differences?
(7) Single images of Form3-GFP expression would be useful to see if there is minor expression in other tissues. The images shown do not support the conclusion that Form3 is expressed in the tendon cytoskeleton belt. Maybe high mag pictures of Form3 and/or Shot may show this?
(8) The paper state there is no embryo phenotype observed for form3 mutants (data not shown). Did the authors look carefully to make sure the 'chain of shortened muscle' phenotype is not present? This could be developmental as it seems unlikely that muscle would unattached and reattach in the larval stages.
(9) P. 17, lines 40-41. The statement 'form3 mutant larvae is indirectly caused by aberrant tendon cell functions' may remove the word 'indirect'.
(10) Beta PS integrin is used to mark the elongated tendon cells, but as the authors state, this protein is expressed on the surface of both muscle and tendon cells. Can the authors use the tendon cell alphaPS1 to confirm? Or modify the claims of using Beta PS integrin as a sole marker for tendon cells?
(11) P. 18, lines 35-38. The following sentence is a bit of an overstatement and could be modified to better state what the data shows - 'Therefore, Form3 appears important specifically for stabilizing the specialized cytoskeletal belt in tendon cells of direct MTJs.'
(12) Images in Figure 8 could benefit from single channel views. Maybe a as supplemental figure?
(13) Include statistical analysis in figure legends where appropriate.

© 2025. Published by The Company of Biologists under the terms of the Creative Commons Attribution License (https://creativecommons.org/licenses/by/4.0/).

(14) In Fig. S3, are the tendon cells marked by phalloidin? I think this should be sr>Tomato as muscles are marked with phalloidin.

Minor points
(1) P. 3, lines 10-11. Please modify 'which transmits the strong muscles forces' to 'which transmits strong muscle forces.'
(2) MTJ and MAS seem to be used interchangeably. If so, please specify earlier that these two terms are considered equal in this case.
(3) P. 17, lines 31-33. Add quantitation for the statement 'The phenotype is less severe as compared to those of the above-described EMS alleles.'
(4) P. 17, lines 40-41. The statement 'form3 mutant larvae is indirectly caused by aberrant tendon cell functions' may remove the word 'indirect'.
(5) P. 17, lines 50-51. 'transheterozygously mutant' is awkward. Please change.
(6) Explain GAP-GFP
(7) Label all muscles that will be examined in Fig. 5A.
(8) Explain and/or define 'on-view.' What about XZ projection?
(9) Explain WT first then mutant situations (Fig. 6)
(10) Explain the differences in Form2 localization for the GFP and HS forms.
(11) 'Phenotypic muscles' is awkward as written in Fig. 2F. Pl

Reviewer 2

SUMMARY OF THE ADVANCE MADE IN THIS PAPER AND ITS POTENTIAL SIGNIFICANCE TO THE FIELD
The identification of novel proteins that maintain tendon cell integrity is impactful, and the findings of this manuscript could enhance our understanding of musculoskeletal dynamics across species.

SUGGESTIONS TO AUTHORS
Major concerns.

1. When is the form3 muscle phenotype first observed and does this coincide with Form3 expression? Specifically, do L1 or L2 larvae express Form3-GFP? Do L1 and L2 larvae muscles show the balled up phenotype? Do the embryonic tissues that express form3 RNA (by in situ including trachea, nervous system) also express Form3-GFP? It seems possible that embryonic tendons express low levels of Form3 that could be better detected with the protein trap than by less sensitive in situ methods.

2. Since the sr>form3.IR experiment is used as a substitute for a classical rescue, the knockdown phenotype could be quantified in more detail. Which muscles were affected and at what frequency?

3. The short muscles in form3 mutants remain connected to tendons as evidenced by BPSintegrin staining (Fig. 5), while other affected muscles attach to each other. How often are these phenotypes observed? How many animals were assayed. Please provide a control larvae labeled with BPSintegrin.

4. Continuing with Fig. 5, it looks like a dorsal oblique muscle has the balled-up phenotype in the posterior segment. How often is this (indirect MAS muscles with short phenotype) observed? There also appear to be breaks or tears in the myofibrils of ventral muscles. Can the authors explain this?

5. Fig. 6 is difficult to understand. One problem is that different scales are shown in mutants and controls. The other problem is that landmarks, like unaffected muscles, are seen is some samples

© 2025. Published by The Company of Biologists under the terms of the Creative Commons Attribution License (https://creativecommons.org/licenses/by/4.0/).

but not in others. Are the dorsal tips of LT muscles shown in controls and ventral tips shown in mutants? This is quite confusing. Since the phenotype is so dramatic the tendons of course look very different between mutants and controls, but helping to orient the reader would clarify the phenotypes. I encourage the authors to develop some diagrams to accompany the figures to further clarify the histology. Also, how many muscles were assayed for each combination of markers and how often were the various mislocalizations observed?

6. Constitutively active Diaphenous expression in tendon cells caused an expansion of the Shot domain, which is explained as an extra round of cell proliferation due to the appearance of structures that look like ectopic nuclei (Fig. S4G). There are two problems with this conclusion: nuclear labeling is not shown and there appear to be long "cables" of Shot localization in a subset of tendons similar to form3 IR. Since there is no wild-type control of Shot/F-actin to easily compare with the CA-Dia tendons, it is difficult to know if there is a CA-Dia phenotype or not. Perhaps a more careful examination of the histology will show there is in fact a CA-Dia phenotype.

Minor points.

1. What is gapGFP (Fig. S2) and why was it used?

2. I'm not aware of muscle-muscle attachments occurring in other mutants. Can the authors comment on whether this phenotype has been reported before and how it might arise.

3. Are there known roles for formins (or inverted formins) in tendons of other species?

FURTHER COMMENTS

The manuscript by Muster et al identifies Formin-3 as a novel regulator of tendon cell integrity. A forward genetic screen uncovered two alleles with muscle defects in 3rd instar larvae that mapped to form3. In-depth phenotyping revealed a subset of muscles in form3 mutants were shorter than wild-type muscles due to stretching of the tendon cells. Interestingly, aAected muscles correlated with type of muscle-tendon attachments. Muscles that make intrasegmental tendon attachments (direct muscle attachments) were aAected while muscles that attached to tendons at segment borders were unaAected. A GFP protein trap line showed form3 is expressed in 3rd instar and adult tendon cells. In 3rd instar larvae, Form3-GFP accumulated at the tips of muscles that make direct attachments but not on muscles that make indirect attachments. To understand the cellular role of Form-3 in tendon cells, the authors generated Form3-HA knock-in lines and found Form3 localizes to F-actin filaments and microtubule arrays in tendon cells.

The identification of novel proteins that maintain tendon cell integrity is impactful, and the findings of this manuscript could enhance our understanding of musculoskeletal dynamics across species. Overall, the data presented are convincing that Form3 plays a critical role in tendon cell integrity. However, some additional insights and clarifications would help strengthen the general conclusions of the manuscript.

Major concerns.

1. When is the form3 muscle phenotype first observed and does this coincide with Form3 expression? Specifically, do L1 or L2 larvae express Form3-GFP? Do L1 and L2 larvae muscles show the balled up phenotype? Do the embryonic tissues that express form3 RNA (by in situ including trachea, nervous system) also express Form3-GFP? It seems possible that embryonic tendons express low levels of Form3 that could be better detected with the protein trap than by less sensitive in situ methods.

© 2025. Published by The Company of Biologists under the terms of the Creative Commons Attribution License (https://creativecommons.org/licenses/by/4.0/).

2. Since the sr>form3.IR experiment is used as a substitute for a classical rescue, the knockdown phenotype could be quantified in more detail. Which muscles were aAected and at what frequency?

3. The short muscles in form3 mutants remain connected to tendons as evidenced by BPSintegrin staining (Fig. 5), while other aAected muscles attach to each other. How often are these phenotypes observed? How many animals were assayed. Please provide a control larvae labeled with BPSintegrin.

4. Continuing with Fig. 5, it looks like a dorsal oblique muscle has the balled-up phenotype in the posterior segment. How often is this (indirect MAS muscles with short phenotype) observed? There also appear to be breaks or tears in the myofibrils of ventral muscles. Can the authors explain this?

5. Fig. 6 is diAicult to understand. One problem is that diAerent scales are shown in mutants and controls. The other problem is that landmarks, like unaAected muscles, are seen is some samples but not in others. Are the dorsal tips of LT muscles shown in controls and ventral tips shown in mutants? This is quite confusing. Since the phenotype is so dramatic the tendons of course look very diAerent between mutants and controls, but helping to orient the reader would clarify the phenotypes. I encourage the authors to develop some diagrams to accompany the figures to further clarify the histology. Also, how many muscles were assayed for each combination of markers and how often were the various mislocalizations observed?

6. Constitutively active Diaphenous expression in tendon cells caused an expansion of the Shot domain, which is explained as an extra round of cell proliferation due to the appearance of structures that look like ectopic nuclei (Fig. S4G). There are two problems with this conclusion: nuclear labeling is not shown and there appear to be long “cables” of Shot localization in a subset of tendons similar to form3 IR. Since there is no wild-type control of Shot/F-actin to easily compare with the CA-Dia tendons, it is diAicult to know if there is a CA-Dia phenotype or not. Perhaps a more careful examination of the histology will show there is in fact a CA-Dia phenotype.

Minor points.

1. What is gapGFP (Fig. S2) and why was it used?

2. I’m not aware of muscle-muscle attachments occurring in other mutants. Can the authors comment on whether this phenotype has been reported before and how it might arise.

3. Are there known roles for formins (or inverted formins) in tendons of other species?

<u>Reviewer 3</u>

SUMMARY OF THE ADVANCE MADE IN THIS PAPER AND ITS POTENTIAL SIGNIFICANCE TO THE FIELD

In this interesting manuscript, the authors investigate the role of a formin called formin 3 during Drosophila larval and tendon development. Specifically, the authors focus on the intra-segmental (or direct) tendons, to which individual muscles attach within a larval segment. They find that novel mutations in formin 3 (form3) specifically result in muscle defects of the lateral transverse muscles (muscles 21-24) and much less in muscles that attach at the segment border. This is a

© 2025. Published by The Company of Biologists under the terms of the Creative Commons Attribution License (https://creativecommons.org/licenses/by/4.0/).

highly interesting and specific muscle phenotype that, to my knowledge, had not been described for other mutants previously.
The authors find that a (non-functional) form3-GFP insertion strongly labels the intra-segmental tendons (with some expression at the segment border tendons as well). Interestingly, the homozygous form3-GFP shows that the short lateral transverse muscle phenotype does not result from a muscle detachment but rather from a severe elongation of the intra-segmental tendon cells, a really beautiful phenotype. These abnormally elongated tendons (also some tendons at the segment border are too long in form 3 -/-) are also seen with integrin markers in the form3 mutant. A comparable phenotype is also found in adult flight muscle-tendon attachments. The authors conclude that form3 is required to make the intra-segmental tendon cells rigid and stretch-resistant against the high muscle forces produce in crawling third instar larvae. Hence, tendons can anchor the muscles close to the cuticle.
Mechanistically, the authors show that form3 is needed to bundle actin and microtubules in the intra-segmental tendons, as in the form3 mutant both filaments are highly elongated, suggesting that this defect is the cause for the elongated tendon phenotype.
To observe where Form3 protein is located, the authors generated a knock-in, tagging the C-term of Form3 with HA. They find that Form3-HA largely labels the center of the actin-rich region of the tendon attachment plate, where is likely organises the actin cytoskeleton.
Together, I find this a beautiful manuscript describing the function of a not well characterised gene with a beautiful novel muscle-tendon phenotype. I only have very minor suggestions.

SUGGESTIONS TO AUTHORS
Minor points:
1. The schemes in Figure 1 are very nice and helpful. However, since the text is not only citing publications to support these schemes but also "and own observations" (page 3), it would be nice to support them with some data, like 3-dimensional reconstruction of the intra-segmental tendon cells. These come then only in Figure 6. Maybe one image could be moved to Figure 1?

2. I find it confusing that the blue actin on Figure 6 has no label. And in the yz-view, actin is in white. It might be better to stick to one colour in the figure.

3. page 10 has "Fly/Manchester, 2015" as erroneous citation?

4. Figure S3 should contain the genotype. Currently, it is only in the figure legend.

5. If the authors want to test that the flight muscle tendon phenotype is force-induced, form3 mutants could be combined with viable but flightless Mhc[10] mutants (PMID 39163855).

First revision

<u>Author response to reviewers' comments</u>

List of responses to the reviewers

(the major changes in the revised resubmission are underlined in the manuscript text (version “Form3 paper text HIGHLIGHTED”)

Reviewer 1:

© 2025. Published by The Company of Biologists under the terms of the Creative Commons Attribution License (https://creativecommons.org/licenses/by/4.0/).

Essential tasks

(1) The title must be modified as the paper does not show that force directly impacts Form3 function and/or expression in tendon cells.
("Formin 3 Confers Cytoskeleton Resilience Against Muscle Tensile Forces in Drosophila Tendon Cells")

While the previous title in our opinion did not imply that muscle force impacts Form3 function or expression, we have modified it to "Formin 3 Stabilizes the Cytoskeleton of *Drosophila* Tendon Cells, thus Enabling them to Resist Muscle Tensile Forces", which puts the primary emphasis on its tendon stabilization function.

(2) Two review papers are used as references in the first intro paragraph. Schweitzer, 2010 is quite old and it would be better for readers to refer the original manuscripts, especially for bidirectional crosstalk between muscles and tendons during embryonic development.

We added 4 original papers and mentioned myotube pathfinding (p. 3, 1st paragraph).

(3) As currently written, the references for the ZPD proteins imply a direct role in embryonic and/or larval muscle- tendon attachments. Wilkin, et al., contains no functional data for Dumpy in this process. Bokel, et al., looks at wing and embryo while Chu and Hayashi examines IFMs. Please modify accordingly as this reviewer is unaware of a direct role for ZPD proteins in larval MAS. Accordingly, can ZPDs be conclusively shown the Fig. 1?

Chu & Hayashi show Dumpy expression and a role of qsm in larval muscle insertion sites (their Fig. 6). Bökel et al. show tonofilament anchoring defects in cuticle at muscle insertion sites in late embryo/L1 stages (their Fig. 5). Therefore we retained this statement and the schematic drawings in fig. 1, but we deleted Wilkin et al. because it only shows late embryonic expression of Dpy.

(4) Please renumber the figures, tables, etc. so they are referred to in order in the manuscript.

We have now ordered the figures (including the newly added ones in the supplement) and tables as they appear in the Results section and put the table mentioned in M&M last.

(5) Add the lethality data (p. 13, lines 39-54) to Table S5. Also, on p. 14, lines 20-24, include a comparison of Em31 in the allelic strength discussion.

We moved these data to the table (now Table 1) and added our lethality data for the alleles Em31, F958, and S265 (all in trans to the deficiency) for mutual comparison to it.

(6) The sequencing results and domain descriptions would be easier to read (p. 15-16) if the domains/protein characteristics were all discussed first followed by the lesions identified. What about a lack of domains in the C- terminus (maybe mention this). Is the lack of a DAD conserved in mammalian formin3 proteins? Is it worth showing comparisons to other organisms due to these differences?

We rearranged the statements in this paragraph such that they follow the order shown in Fig. 3, as suggested (p.16, top). Regarding the DAD domain, we modified our statement by emphasizing **"In contrast to mammalian Form3 proteins,** *Drosophila* Form3 lacks a DAD domain" (p. 15, bottom).

© 2025. Published by The Company of Biologists under the terms of the Creative Commons Attribution License (https://creativecommons.org/licenses/by/4.0/).

As domain comparisons with other species are shown in the cited review article, we feel it is unnecessary and possibly distracting to show these again.

(7) Single images of Form3-GFP expression would be useful to see if there is minor expression in other tissues. The images shown do not support the conclusion that Form3 is expressed in the tendon cytoskeleton belt. Maybe high mag pictures of Form3 and/or Shot may show this?

These points refer to the previous version of Fig. 4, which we have replaced by new panels that focus on tendon cell expression but not subcellular localization (including 1st and 2nd instar as queried by reviewer 2). We omitted statements about tendon cytoskeletal belt localization here as this issue is best resolved with higher magnifications and 3D analyses as done for Fig. 7. Regarding the expression in other tissues, we now describe the temporal and tissue expression of both Form3-GFP and Form3-HA during embryogenesis (in part, to show absence of expression in embryonic tendon cells as queried by reviewer 2) (new Fig. S2).

(8) The paper state there is no embryo phenotype observed for form3 mutants (data not shown). Did the authors look carefully to make sure the 'chain of shortened muscle' phenotype is not present? This could be developmental as it seems unlikely that muscle would unattached and reattach in the larval stages.

We agree that a priori this phenotype is unexpected. To exclude that this is a developmental phenotype already present in embryos, we repeated embryo stainings with mutants, using both MHC and shaker-GFP (present on the mutant chromosome) as muscle markers. Careful analysis of late stage mutant embryos showed that their muscle pattern is completely normal, including the absence of contracted muscles. However, live imaging for shaker-GFP clearly showed that muscle phenotypes first appear in 1st instar larvae, which fits to our new data showing that Form3 expression initiates at the transition from late embryonic to 1st instar larval stages. These phenotypic analyses in embryos and 1st to 3rd instar larvae are now presented in the new Fig. S3.

(9) P. 17, lines 40-41. The statement 'form3 mutant larvae is indirectly caused by aberrant tendon cell functions' may remove the word 'indirect'.

Removed.

(10) Beta PS integrin is used to mark the elongated tendon cells, but as the authors state, this protein is expressed on the surface of both muscle and tendon cells. Can the authors use the tendon cell alphaPS1 to confirm? Or modify the claims of using Beta PS integrin as a sole marker for tendon cells?

Unfortunately, we did not have any alphaPS1 antibodies available. However, we used several different tendon cell markers in other contexts, many of which are shown later (including the two differently tagged Form3 versions, several stripe-GAL4-driven markers, beta1-Tubulin, and others). Thus, we don't think alphaPS1 would add any essential information to the new alphaPS2 stainings now shown in Fig. 5.

(11) P. 18, lines 35-38. The following sentence is a bit of an overstatement and could be modified to better state what the data shows - 'Therefore, Form3 appears important specifically for stabilizing the specialized cytoskeletal belt in tendon cells of direct MTJs.'

We modified the sentence to: "…important specifically for stabilizing the **mechanical integrity of the tendon cells** of direct MTJs." (p. 19 top)

© 2025. Published by The Company of Biologists under the terms of the Creative Commons Attribution License (https://creativecommons.org/licenses/by/4.0/).

(12) Images in Figure 8 could benefit from single channel views. Maybe a as supplemental figure?

We assume the reviewer refers to Fig. 6 as there was no Fig. 8. As suggested, we composed a new supplemental figure (Fig. S7) showing separate channels for tendon cell-driven, F-Actin-bound RFP plus Zipper (as seen in Fig. 6A, B in combined channels), as well as the single channel for beta1-Tubulin (as seen in Fig. 6F in combined channels). We felt that showing all panels from Fig.6 as single channels would have made this new Figure too unwieldy and condensed on the page.
The new Fig. S7 also includes clarifying information requested in point 5 of reviewer 2.

(13) Include statistical analysis in figure legends where appropriate.

We performed additional statistics and the respective data are now shown as graphs in Fig. 2 and as a new Fig. S4.

(14) In Fig. S3, are the tendon cells marked by phalloidin? I think this should be sr>Tomato as muscles are marked with phalloidin.

Thank you for catching this error. We changed it to: "(red, stained for tractin-Tomato with anti-RFP)" (now Fig. S8).

Minor points

(1) P. 3, lines 10-11. Please modify 'which transmits the strong muscles forces' to 'which transmits strong muscle forces.'

Done.

(2) MTJ and MAS seem to be used interchangeably. If so, please specify earlier that these two terms are considered equal in this case.
In the intro on p. 3, we now write: "myotendinous junctions **(also referred to as muscle attachment sites (MAS))**"

(3) P. 17, lines 31-33. Add quantitation for the statement 'The phenotype is less severe as compared to those of the above-described EMS alleles.'

Because this statement was based on our overall impression and we did not manage to generate sufficient quantitative data for it, we have deleted it.

(4) P. 17, lines 40-41. The statement 'form3 mutant larvae is indirectly caused by aberrant tendon cell functions' may remove the word 'indirect'.

Done.

(5) P. 17, lines 50-51. 'transheterozygously mutant' is awkward. Please change.

We now write out the exact genotype.

(6) Explain GAP-GFP

We have added the explanation to the stock name in M&M (*P{w[+mC]=UAS-gapGFP}AC1* (BL-4522)

© 2025. Published by The Company of Biologists under the terms of the Creative Commons Attribution License (https://creativecommons.org/licenses/by/4.0/).

(expresses
GFP tagged with the myristylation sequence from GAP43) and, likewise, to the legend in Fig S6.

(7) Label all muscles that will be examined in Fig. 5A.

Fig. 5 was replaced and we labeled all relevant muscles seen in new Fig. 5. We also added more muscle labels to the other figures for reference (e.g., new Fig. S7).

(8) Explain and/or define 'on-view.' What about XZ projection?

We decided to change it into “top-down view” and defined it at first appearance: “top-down view (i.e., Z-stack projection of several XY scans)” and hope this sounds clearer with it being too technical.

(9) Explain WT first then mutant situations (Fig. 6)

We explain WT first and then mutant for each antibody combination. We do not think it would be helpful to first explain the WT situations for all antibody combinations and thereafter all mutant situations for the different antibody combinations because this would impede direct juxtaposition.

(10) Explain the differences in Form2 localization for the GFP and HS forms.

We expanded the previous description a little (“Form3-GFP overlaps with Form3-HA in these fibrils, but unlike Form3-HA, which is enriched in central areas of the attachment plate (Fig. 7A2, arrowheads), form3-GFP is also present in the apical and basal areas.” (p. 22) and added arrowheads to the borders of the Form3-HA domain in Fig. 7.

(11) 'Phenotypic muscles' is awkward as written in Fig. 2F. Pl

Changed to ‘abnormal’

Reviewer 2:
Major concerns.

1. When is the form3 muscle phenotype first observed and does this coincide with Form3 expression? Specifically, do L1 or L2 larvae express Form3-GFP? Do L1 and L2 larvae muscles show the balled up phenotype? Do the embryonic tissues that express form3 RNA (by in situ including trachea, nervous system also express Form3-GFP? It seems possible that embryonic tendons express low levels of Form3 that could be better detected with the protein trap than by less sensitive in situ methods.

We performed new experiments to address all these points. As already stated above for reviewer 1 (8), we re- examined the muscle pattern in *form3* mutant embryos at late stages and did not detect any abnormalities (new Fig. S3A - B”).
Via live imaging of Form3-GFP in larvae, we could show that Form3 starts to be expressed in 1st instar larvae in tendon cells, and continues this expression during 2nd and 3rd instar (new Fig. 4E, F).
Via live imaging of shaker-GFP in larval muscles, we could show that the shortened muscle phenotype appears already in 1st instar larvae, and at least some aspects of it get stronger

© 2025. Published by The Company of Biologists under the terms of the Creative Commons Attribution License (https://creativecommons.org/licenses/by/4.0/).

until 3rd instar (new Fig. S3C – N; statistics in new Fig. S4).
With HA-stainings of form3-HA embryos we found a single stage 17 embryo that showed tendon cell expression (new Fig. S2R); all others and all form-GFP embryos stained with anti-GFP were negative in these cells (new Fig. S2). The exceptional positive embryo may have been close to an L1 larval stage and perhaps had a damaged cuticle that allowed antibody penetration to be stained. In summary, these new data show that Form3 expression initiates in tendon cells at the transition between embryonic stage 17 and larval L1, which closely matches the appearance of the muscle phenotype.
In addition, we document the temporal and tissue-specific expression of Form3-GFP and Form3-HA throughout embryogenesis, which shows expression in the tissues previously identified by RNA I situs, as well as in some additional tissues that had not been reported. As also stated in the text, both in vivo tagged versions are expressed almost identically in the trachea, cns, fat body, pericardial cells, and the anal plate (new Fig. S2).

2. Since the sr>form3.IR experiment is used as a substitute for a classical rescue, the knockdown phenotype could be quantified in more detail. Which muscles were affected and at what frequency?

We added to the legend for Fig. S9A: “Among 20 scanned LTMs and five M18, 100% were severely affected.”

3. The short muscles in form3 mutants remain connected to tendons as evidenced by BPSintegrin staining (Fig. 5), while other affected muscles attach to each other. How often are these phenotypes observed? How many animals were assayed. Please provide a control larvae labeled with BPSintegrin.

We replaced Fig. 5 with newly generated data, including a control larva, and moved the previous Fig. 5 into the supplement (now Fig. S5). The statistical analysis is provided in new Fig. 5D and the number of abnormalities and of analyzed hemisegments is provided in Results (p. 18) and in Fig. 5 legend.

4. Continuing with Fig. 5, it looks like a dorsal oblique muscle has the balled-up phenotype in the posterior segment. How often is this (indirect MAS muscles with short phenotype) observed? There also appear to be breaks or tears in the myofibrils of ventral muscles. Can the authors explain this?

This is now Fig. S5A. The dorsal oblique muscle referred to was very likely damaged during the filet preparation, which is not unusual because the larvae are cut open with scissors along the dorsal midline. The intensity irregularities in some groups of ventral muscles are an artifact with the Zeiss Apotome that is sometimes obtained in areas with high tissue densities. We added the information on the use of the Apotome to the figure legend (as all other images were obtained with confocal imaging, as described in M&M).

5. Fig. 6 is difficult to understand. One problem is that different scales are shown in mutants and controls. The other problem is that landmarks, like unaffected muscles, are seen is some samples but not in others. Are the dorsal tips of LT muscles shown in controls and ventral tips shown in mutants? This is quite confusing. Since the phenotype is so dramatic the tendons of course look very different between mutants and controls, but helping to orient the reader would clarify the phenotypes. I encourage the authors to develop some diagrams to accompany the figures to further clarify the histology. Also, how many muscles were assayed for each combination of markers and how often were the various mislocalizations observed?

© 2025. Published by The Company of Biologists under the terms of the Creative Commons Attribution License (https://creativecommons.org/licenses/by/4.0/).

We concede that the data in Fig. 7 need a bit of diligence in order to be interpreted fully and thus have followed some of the suggestion made by the reviewer to mitigate this. While we have not greatly changed the figure itself, we did add information in each panel on whether the dorsal or the ventral tips of the respective muscles are shown. Except for Fig. 7B, all other panels show the dorsal tips and we wanted to keep Fig. 7B, because it shows a very nice example of the changes (and because for the LT muscles, their dorsal and ventral attachments look the same). We also added the phalloidin color codes on the bottom of the figure. Importantly, we generated a new Figure (Fig. S7), which includes some of the clarifying features suggested above in direct reference to Fig. 6. First, we show the schematic outlines of the muscles and tendon cells from Fig. 6A (control) and B (mutant), which makes it easier to understand the situation, especially for this one case where the two different ends of the muscles are compared. Second, we also show an overview of the abnormal muscles with their stretched tendon cells from Fig. 6B (and S7B) in the context of the neighboring muscles within the entire hemisegment (taken at 20x magnification) and marked many muscles with numbers (Fig. 7C). Analogous low magnification images to show the neighboring context of the affected muscles and tendons for the marker combinations presented in Fig. 6D and H are also included (Fig. S7D and F, respectively). Together with the schematic outlines, as well as the separate channels shown (Fig. S7A -B", E; see response to Reviewer 1) these additions should greatly improve the interpretation of the data in Fig. 7.

The reason for the different magnifications in the controls vs. mutants was that we wanted to scan and present the attachments in the controls at maximal resolutions, but due to the severe stretching of the tendon cells in the mutants they occupy more space and had to be scanned at a somewhat lower magnification in order to fit.

We added the information on the number of muscles assayed for each combination to M&M (Microscopy). Three to five mutant muscle/tendon cell arrangements were scanned at 63x for each combination (which required hundreds of scanning hours to capture the full Z information and the three sequentially scanned channels). In addition, we scanned between ca. 30 and 120 muscles at 20x or 10x magnification. The results were principally reproducible, but obviously the degrees of tendon cell stretching varied and the lower magnifications afforded less detail.

6. Constitutively active Diaphenous expression in tendon cells caused an expansion of the Shot domain, which is explained as an extra round of cell proliferation due to the appearance of structures that look like ectopic nuclei (Fig. S4G). There are two problems with this conclusion: nuclear labeling is not shown and there appear to be long "cables" of Shot localization in a subset of tendons similar to form3 IR. Since there is no wild-type control of Shot/F- actin to easily compare with the CA-Dia tendons, it is difficult to know if there is a CA-Dia phenotype or not. Perhaps a more careful examination of the histology will show there is in fact a CA-Dia phenotype.

In our opinion, the interpretation of two cells being present at muscle ends is clear even without nuclear labeling, because the nuclei devoid of cytoplasmic Shot can be clearly identified. Because there was an empty space in the Figure (now Fig. S9), we added an enlarged panel in which we merged fewer Z planes and show Shot in white, which shows the nuclei even more clearly (Fig. S9G'). We thought it is worth mentioning this phenotype although it is not directly relevant to our main topic. In Fig. S9G legend we added: (see Fig. 3A for control).

The muscle referred to was the only muscle end with Shot streaks observed. We went back to the individual Z planes and found that these are not associated with a retracted muscle end (instead, there is some interdigitation with myofibrils). Because this was only seen in this single case, we do not think it is warranted to make a point out of it. However, if the reviewer disagrees, we can certainly add a higher magnification view with fewer merged Z planes of this muscle end.

© 2025. Published by The Company of Biologists under the terms of the Creative Commons Attribution License (https://creativecommons.org/licenses/by/4.0/).

Minor points.

1. What is gapGFP (Fig. S2) and why was it used?

As noted for reviewer 1 (6), we have added the explanation to the stock name in M&M (*P{w[+mC]=UAS- gapGFP}AC1* (BL-4522) **(expresses GFP tagged with the myristylation sequence from GAP43)** and, likewise, to the legend in (now) Fig S6. It was used in hopes of specifically labeling the epidermal tendon cell membranes, but as seen in the figure, there is also significant cytoplasmic labeling.

2. I'm not aware of muscle-muscle attachments occurring in other mutants. Can the authors comment on whether this phenotype has been reported before and how it might arise.

We agree that this is an unexpected phenotype. On p. 18, we "speculate that these peculiar arrangements are the result of secondary processes occurring after the complete rupture of some elongated tendon cells, when the presence of adhesive material at the membranes of muscle ends, including integrins, extracellular matrix, and fragmented tendon cell material, may promote ectopic attachments of released muscle ends. However, due to the impossibility of live imaging in the moving larvae, the exact processes establishing these abnormal muscle configurations upon muscle detachments are very difficult to ascertain." This phenotype has not been reported in other muscle attachment mutants, perhaps because in strong cases (e.g., *short stop*) the muscles detach already in late embryos and there is embryonic lethality, which may not allow sufficient time for the detached muscles to reorganize. In mutants with weaker tendon cell stretching, the muscles in larvae may not completely detach, thus preventing their reorganization.

3. Are there known roles for formins (or inverted formins) in tendons of other species?

There are no known roles for any formins in tendons of other species and we added to the last sentence "it would be intriguing to determine whether INF2 is required for generating the rigidity of the cytoskeletal arrays of these support cells **(and likewise, to vertebrate tendons)** as well."

Reviewer 3:

Minor points:

1. The schemes in Figure 1 are very nice and helpful. However, since the text is not only citing publications to support these schemes but also "and own observations" (page 3), it would be nice to support them with some data, like 3- dimensional reconstruction of the intra-segmental tendon cells. These come then only in Figure 6. Maybe one image could be moved to Figure 1?

We followed the suggestion and added a 3D reconstructed confocal image of a side view to the left of the schematic, similar to the one shown in Fig. 6 A1.

2. I find it confusing that the blue actin on Figure 6 has no label. And in the yz-view, actin is in white. It might be better to stick to one colour in the figure.

We added the color codes for phalloidin on the bottom of Fig. 6 and to Fig. S7. The reason for choosing either blue or white is because (1) in Z merges of top-down views, white tends to obscure the color signals at the same positions when merged and (2) white highlights the

© 2025. Published by The Company of Biologists under the terms of the Creative Commons Attribution License (https://creativecommons.org/licenses/by/4.0/).

muscles more clearly than blue, and in merged side views there is generally no spatial overlap between white muscle and colored tendon cell signals.

3. *page 10 has "Fly/Manchester, 2015" as erroneous citation?*

This is a figshare article, and they request the following citation on this website: Fly, Man (2015). How to make filet dissections of Drosophila larvae - a training movie.mov. figshare. Media. https://doi.org/10.6084/m9.figshare.1344809.v2

So instead of writing Manchester out as before, we now use exactly the above format (although we agree that it sounds a bit funny, which may be their intention).

4. *Figure S3 should contain the genotype. Currently, it is only in the figure legend.*

Done (now Fig. S9)

5. *If the authors want to test that the flight muscle tendon phenotype is force-induced, form3 mutants could be combined with viable but flightless Mhc[10] mutants (PMID 39163855).*

We agree that this would be a neat experiment but it would have been but quite lengthy by the time the flies arrive and the double mutants are made (and include the risk that healthy double mutant lines cannot be established). As we now know that there is a phenotype in 1st instar larvae, it would be preferable to suppress the L1 tendon phenotype with genetic situations that eliminate muscle contractions already in the embryo. Alas, we did not do that (plus, we think the results are predictable).

Second decision letter

MS ID#: jcs.263543R1

MS Title: Formin 3 Stabilizes the Cytoskeleton of Drosophila Tendon Cells, thus Enabling them to Resist Muscle Tensile Forces

Authors: Manfred Frasch; Helena Pissarek; Leanna H. Frasch; Hermann Aberle; Na Huang

Article Type: Research Article

Dear Dr Frasch,

I am happy to tell you that your manuscript has been accepted for publication in Journal of Cell Science, pending standard publication integrity checks.

© 2025. Published by The Company of Biologists under the terms of the Creative Commons Attribution License (https://creativecommons.org/licenses/by/4.0/).
